# Patterns of gray and white matter functional networks involvement in glioblastoma patients: indirect mapping from clinical MRI scans

**DOI:** 10.3389/fneur.2023.1175576

**Published:** 2023-06-20

**Authors:** Giulio Sansone, Lorenzo Pini, Alessandro Salvalaggio, Matteo Gaiola, Francesco Volpin, Valentina Baro, Marta Padovan, Mariagiulia Anglani, Silvia Facchini, Franco Chioffi, Vittorina Zagonel, Domenico D’Avella, Luca Denaro, Giuseppe Lombardi, Maurizio Corbetta

**Affiliations:** ^1^Department of Neuroscience, University of Padova, Padova, Italy; ^2^Padova Neuroscience Center (PNC), University of Padova, Padova, Italy; ^3^Division of Neurosurgery, Azienda Ospedaliera Università di Padova, Padova, Italy; ^4^Academic Neurosurgery, Department of Neurosciences, University of Padova, Padova, Italy; ^5^Department of Oncology, Oncology 1, Veneto Institute of Oncology IOV-IRCCS, Padova, Italy; ^6^Neuroradiology Unit, University Hospital of Padova, Padova, Italy; ^7^Venetian Institute of Molecular Medicine (VIMM), Fondazione Biomedica, Padova, Italy

**Keywords:** glioblastoma, functional gray matter networks, functional white matter networks, MRI, overall survival, patterns

## Abstract

**Background:**

Resting-state functional-MRI studies identified several cortical gray matter functional networks (GMNs) and white matter functional networks (WMNs) with precise anatomical localization. Here, we aimed at describing the relationships between brain’s functional topological organization and glioblastoma (GBM) location. Furthermore, we assessed whether GBM distribution across these networks was associated with overall survival (OS).

**Materials and methods:**

We included patients with histopathological diagnosis of IDH-wildtype GBM, presurgical MRI and survival data. For each patient, we recorded clinical-prognostic variables. GBM core and edema were segmented and normalized to a standard space. Pre-existing functional connectivity-based atlases were used to define network parcellations: 17 GMNs and 12 WMNs were considered in particular. We computed the percentage of lesion overlap with GMNs and WMNs, both for core and edema. Differences between overlap percentages were assessed through descriptive statistics, ANOVA, post-hoc tests, Pearson’s correlation tests and canonical correlations. Multiple linear and non-linear regression tests were employed to explore relationships with OS.

**Results:**

99 patients were included (70 males, mean age 62  years). The most involved GMNs included ventral somatomotor, salient ventral attention and default-mode networks; the most involved WMNs were ventral frontoparietal tracts, deep frontal white matter, and superior longitudinal fasciculus system. Superior longitudinal fasciculus system and dorsal frontoparietal tracts were significantly more included in the edema (*p* < 0.001). 5 main patterns of GBM core distribution across functional networks were found, while edema localization was less classifiable. ANOVA showed significant differences between mean overlap percentages, separately for GMNs and WMNs (*p*-values<0.0001). Core-N12 overlap predicts higher OS, although its inclusion does not increase the explained OS variance.

**Discussion and conclusion:**

Both GBM core and edema preferentially overlap with specific GMNs and WMNs, especially associative networks, and GBM core follows five main distribution patterns. Some inter-related GMNs and WMNs were co-lesioned by GBM, suggesting that GBM distribution is not independent of the brain’s structural and functional organization. Although the involvement of ventral frontoparietal tracts (N12) seems to have some role in predicting survival, network-topology information is overall scarcely informative about OS. fMRI-based approaches may more effectively demonstrate the effects of GBM on brain networks and survival.

## 1. Background

Glioblastoma (GBM) is the most common primary malignant tumor of the central nervous system in the adult population. The incidence rate for GBM is 3–5 people per 100.000 per year (1–4). Despite advances in neurosurgery, neuro-oncology and radiotherapy, overall survival (OS) at 5 years is currently about 6.8%, with a median OS around 15 months (3, 4). The main prognostic factors are: age at diagnosis, performance status before surgery, extent of resection, eligibility to radio- or chemotherapies, O-6-methylguanine-DNA methyltransferase promoter (MGMT) methylation and gender (5, 6). Interestingly, pre-surgical GBM size does not predict patient survival (7).

Concerning their anatomical distribution, GBMs are thought to originate from neural stem cells within the so-called “subventricular zones” (8–10), from which they putatively grow and spread through the white matter (WM) of frontal, temporal and parietal lobes, disrupting the overlaying gray matter (GM) (11–13). On the other hand, occipital and infratentorial localizations are much less frequent. Tumor location might be associated with a relatively worse or better prognosis, depending on the extent of tumor resection allowed by the “neurological eloquence” of that region (7).

Previous neuroradiological studies have shown that brain tumors cause not only structural but also functional alterations in brain networks (14–18), in both ipsilesional and contralesional hemispheres (19, 20). Resting-state functional MRI (rs-fMRI) studies have identified a small number of GM functional networks (GMNs) based upon the temporal correlation of the blood oxygenation level dependent (BOLD) signal between distinct cortical regions. Yeo et al. (21) proposed a hierarchical parcellation of the brain cortex into 7 main cortical GMNs: visual (VIS), somatomotor (SMN), dorsal attention (DAN), ventral-attention (VAN), limbic (LMB), frontoparietal (FPN) and default-mode (DMN) networks. These could be furtherly fractionated into 17 sub-networks, of which somatomotor A, somatomotor B, peripheral vision and central vision are predominantly local networks confined to sensory and motor cortices. The other networks are more distributed across multiple lobes, are related to cognitive functions and are known as associative networks. Interestingly, rs-fMRI studies showed that WM and GM exhibit similar low-frequency signal powers, moreover in task-related fMRI studies it was found that external stimuli could reliably induce a hemodynamic response within the WM, with a profile similar to that observed in GM, though with a smaller peak amplitude (22). The study of WM fMRI signals in neuro-oncology is still widely unexplored and its relevance is potentially very high since GBM is predominantly a WM disease. As an example, some authors have found decreased functional connectivity with DMN in the corpus callosum of glioma patients, potentially explained by tumor-dependent Wallerian degeneration (18). Notably, functional atlases of WM have been defined: in particular, Peer et al. (23) showed the existence of 12 WM functional networks (WMNs). Half of these showed good anatomical correspondence with structural WM tracts, whereas the remaining half simultaneously corresponded to multiple tracts, presumably allowing coordinated activity across multiple GMNs. Furthermore, these were subdivided into superficial WMNs, correlated with established GMNs, and deep WMNs, which do not show such strong correlations and have been postulated to represent the putative means of communication between different GMNs (23). Atlases of resting state fMRI-derived GMNs and WMNs can be currently used for mapping purposes (21, 23).

GBMs are not uniformly distributed across brain functional networks and larger tumors usually encompass both WM and GM. Mandal et al. (13) have recently discovered that gliomas are prevalent within the aforementioned Yeo’s associative networks and areas harboring stem-like brain cells. They also found that functional connectivity measures [based on Miller’s connectome (24)], such as nodal strength, as well as cellular and genetic data explained about 58% of the variance in glioma distribution frequency. In another study, the same authors used independent component analysis to decompose low- and high-grade glioma lesions into 3 principal areas of co-lesioned brain regions (“lesion covariance networks” or “LCNs”), which showed anatomical correspondence to different structural WM tracts and functional connectivity networks [obtained from Miller et al. (24)]. The differences in OS that they found between LCNs, however, were mainly driven by molecular determinants, rather than glioma distribution (25). Recently, a network-based anatomical approach has been proposed for the classification of brain tumors in relation to the cognitive outcome (26).

As for fMRI studies, Liu et al. (27) for the first time implemented fMRI data into the prediction of glioma patient survival, discovering that functional connectivity-derived features increased the accuracy of patient survival prediction. Other rs-fMRI studies showed that OS correlated with specific patterns of BOLD synchronization between tumor core and distant brain regions (28, 29). fMRI studies have the advantage of directly measuring the impact of GBMs on brain functional organization. Moreover, BOLD-signal may also be related to the tumoral neoangiogenesis (30).

Despite recent advances, much remains to be learned about the impact of brain tumors on the brain’s functional networks and their relationship to survival. Advanced MRI studies are costly, time-consuming and not always feasible, especially for large-scale studies or in clinical practice. Hence, the aim of the present study was to use conventional clinical MRI scans to quantify the spatial relationships between GBM lesions and brain’s functional organization, in terms of relative overlap of the neoplastic core and perilesional edema region with both Yeo’s 17 GMNs and Peer’s 12 WMNs, without using fMRI-derived functional connectivity data. Moreover, we aimed at exploring differences between distinct tumor-network overlap percentages, as well as identifying potential patterns of GBM distribution across functional networks. Lastly, we investigated whether the extent of the overlap between core or edema regions and specific GMNs or WMNs improves the prediction of patient survival, in addition to the known clinical-prognostic factors. Since we used anatomical MRI images, our approach may be reproducible in a clinical setting. As compared to Mandal et al.’s (13) study, we used a 17-network parcellation to increase the specificity of GBM to GMNs relationships. Furthermore, to our knowledge, this is the first study to quantify the spatial relationship between GBM lesions and WMNs. Finally, this study is the first to investigate potential links among GBM perilesional edema, functional connectivity networks and patient survival, as edema has been shown to harbor valuable information in previous studies (31). Overall, the present study investigates the spatial relationships between GBM and functional networks, in the wake of an emerging field called “cancer neuroscience.”

## 2. Materials and methods

### 2.1. Patients

This retrospective study was conducted on a cohort of patients with a histologically confirmed, newly diagnosed GBM, IDH wild-type, according to the WHO 2021 classification (32). The inclusion criteria were: (1) a histologically confirmed, newly diagnosed GBM, IDH wild-type; (2) the availability of presurgical MRI acquisition, which had to include T2w, FLAIR, pre- and post-contrast T1w sequences; (3) availability of OS data. The exclusion criteria were: GBM recurrence, MRI acquisition with a low magnetic field scanner (magnetic lower than 1.5 T), lack of axial plane acquisition in at least one among FLAIR, pre- and post-contrast T1w sequences, the presence of macroscopic artifacts in MR structural images, and radiologic evidence of previous brain diagnostic or therapeutic invasive procedures (e.g., stereotactic biopsy). For each patient, the following additional clinical, surgical and prognostic variables were recorded: age, gender, Stupp protocol, radicality of surgical resection (biopsy, partial resection and gross total resection), ECOG performance status and MGMT promoter methylation status.

The study was approved by the ethical committee of the Province of Padua (Comitato Etico per la Sperimentazione Clinica della Provincia di Padova n. 70n/AO/20). The study was performed in accordance with the Declaration of Helsinki and its latest amendments.

### 2.2. Preprocessing of MR images

Structural images were pre-processed before manually delineating the tumor volume. Preprocessing included image bias field correction (33) and skull stripping (34). Structural images were then coregistered to the pre-contrast T1w of the patient to improve the segmentation of the tumor. Manual segmentation was performed in the native space using the ITK-Snap toolbox version 3.8.0^1^ (35) slice-by-slice by a neurology resident and a neurology intern (GS and MG) and checked by an experienced neurologist (AS) and neuroradiologist (MA). The following areas were segmented into two regions of interest (ROI) for each tumor: tumor core (including areas of necrosis, contrast-enhancing tumor or CET and non-contrast-enhancing tumor or nCET) and edema. The segmentations were performed in a step-wise manner, starting from CET, then the necrosis, the edema and, eventually, nCET. The criteria used to differentiate the last two regions were the following (36, 37): edema typically has a “finger-like appearance,” extends concentrically around CET, is characterized by predominant WM involvement, relative “sparing” of subcortical GM nuclei, possible extension along the internal or external capsule and diffuse/generalized mass-effect. Moreover, edema tends to show a marked T2/FLAIR hyperintensity, often fading towards the periphery. Conversely, nCET is characterized by extension beyond CET margin with an eccentric appearance, involves GM and WM more equally (including subcortical GM nuclei) and determines a more localized mass effect, with associated anatomical distortion. Furthermore, T2/FLAIR hyperintensity is relatively milder, as compared with edema. CET, necrosis and nCET (if applicable) were labeled as “core.” Lesions were subsequently normalized through the “virtual brain grafting” approach (38). This approach was chosen based on the size of brain tumors. Usually, brain lesions are normalized through a cost functional masking approach, but this might result in lower quality for large lesions (39) as in our case. The adopted approach generates a donor brain template using the native non-lesioned hemisphere and one hemisphere from a synthetic template brain image (38). For each subject, the donor brain was registered to the MNI space using the Advanced Normalization Tools (40). The transformation matrix was finally applied to the lesion masks using a *nearest neighbor* interpolation approach and resampled to a 1x1x1 mm space.

### 2.3. Tumor-networks overlap computation

For GMNs, Yeo’s parcellation (21) was employed, including the following 17 subnetworks: central vision, peripheral vision, somatomotor A, somatomotor B, dorsal attention A, dorsal attention B, salient ventral attention A, salient ventral attention B, limbic A, limbic B, control network A, control network B, control network C, default mode network A, default mode network B, default mode network C and temporo-parietal networks. We also included deep GM nuclei (basal ganglia and thalami) and hippocampi from Harvard-Oxford subcortical atlas (41) as 18th and 19th parcels for subsequent analyses (i.e., the computation of GM overlap percentages). For WMNs, Peer’s parcellation (23) was used, including the following 12 networks: cingulum and associated tracts (N1), uncinate and middle temporal lobe tracts (N2), sensorimotor superficial WM system (N3), forceps minor system (N4), superior longitudinal fasciculus system (N5), visual superficial WM system (N6), inferior longitudinal fasciculus system (N7), inferior corticospinal tract (N8), posterior cerebellar tracts (N9), dorsal frontoparietal tracts (N10), deep frontal WM (N11), ventral frontoparietal tracts (N12). For each normalized lesion we computed the percentage of overlap with each network, independently for the WMNs and GMNs, as an expression of the ratio between the number of lesion voxels encompassing a specific network and the number of lesion voxels within a specific tissue, that is GM for GMNs and WM for WMNs. Hence, each overlap percentage represents the mask voxels overlapping a specific network, normalized for the mask voxels overlapping all networks of the same tissue (WM or GM). Such computation was performed both with the GBM core and the edema separately. Other overlap percentages were also computed: “not normalized” overlap percentages were calculated as the ratio between the number of lesion voxels encompassing a specific network and the total number of lesion voxels, for core and edema separately; “alternative” overlap percentages were calculated as the ratio between the number of lesion voxels encompassing a specific network and the total number of that network’s voxels; these additional types of overlap percentages were also included in multiple linear regression models (see Supplementary Tables S3, S4).

For each tumor and tissue mask (core and edema) an in-house Python script was written for the following steps: (i) uploading nifti files conveying mask information as vector array; (ii) uploading Peer’s WM and Yeo’s GM nifti atlases in the same vector space (atlas vectors express specific values for each network); (iii) computing the sum of tumor-mask voxels encompassing each vector-network value; (iv) computing the overlap percentages defined above. All the procedure was run through an ASUS TUF Dash F15 machine (12th Gen Intel(R) Core (TM) i7-12650H 2.30 GHz) running on a Ubuntu 20.04.6 LTS (Focal Fossa) environment.

### 2.4. Statistical analyses

Three levels of analyses were performed: (i) descriptive statistics of network involvement through analysis of variance (ANOVA, not including subcortical GM nuclei) to assess differences between networks, independently for WMNs/GMNs and core/edema, as well as with post-hoc comparisons, (ii) assessment of the mutual relationships between the computed overlap percentages through Pearson’s correlations and canonical correlation analysis, a machine learning approach used to measure the association between two sets of variables (performed according to our previous paper (42)); (iii) assessment of the relationships between OS and GBM distribution across functional networks, through linear and non-linear regression tests. For multiple linear regression, independent quantitative and ordinal variables were z-scored preliminarily. These analyses were performed between OS (dependent variable) and each of the following groups of overlap percentages (independent variables), separately: (1) overlap between the GBM core and GMNs, (2) between GBM edema and GMNs, (3) between the GBM core and WMNs and (4) between GBM edema and WMNs. Multiple linear regression analyses were also performed including the following regressors, either with and without the network overlap percentages: age, ECOG performance status, Stupp protocol, radicality of surgical resection (total, subtotal, biopsy), MGMT promoter methylation status, presurgical lesion (core or edema) volume. In addition to the aforementioned analyses, we also investigated relationships between network overlap percentages and the radicality of surgery, through Pearson’s correlation tests. The significance level (alpha) was set to 0.05 and Bonferroni corrections were applied to multiple comparisons and correlations.

Moreover, we performed a non-linear regression analysis by means of the Boruta algorithm, designed to find a subset of features that are relevant to a given classification/regression task (43). The core algorithm behind it is random forests, a methodology able to find non-linear relationships between the dependent and independent variables.

## 3. Results

**Figure 1:**
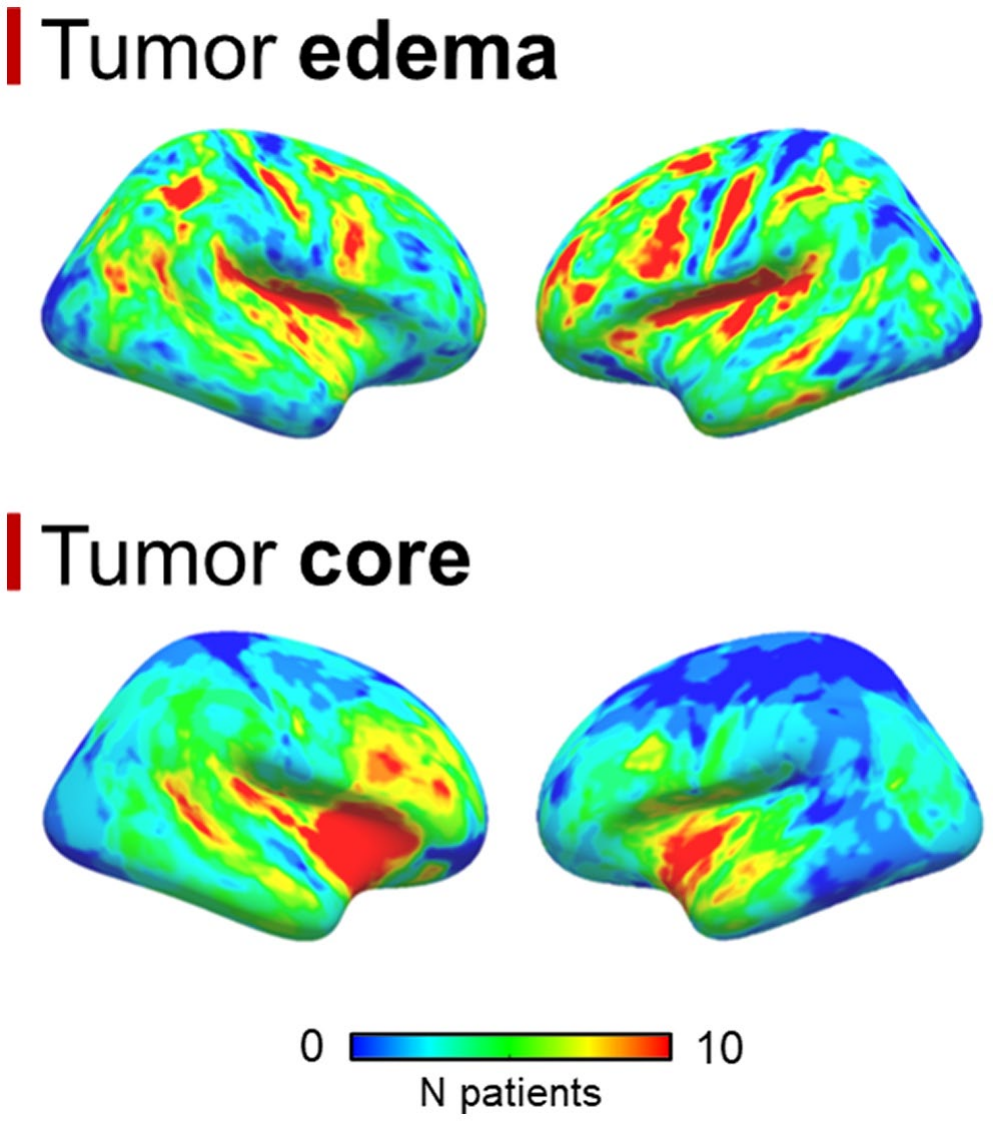
Surface space-projected distribution frequency maps for the GBM core and the edema.

A total of 99 patients were enrolled, 70 were males, the median age was 62 years (interquartile range = 17 years); the median OS was 12.7 months (interquartile range = 15.4 months). No statistically significant differences were found between overlap percentages among age groups (Bonferroni-corrected *p*-values >0.05). Out of 99 patients, 85 had a pre-surgical 3 T MRI study, while 14 had a 1.5 T MRI scan. 86 patients had 3D pre-, post-contrast T1 and FLAIR sequences, while the remaining 13 had at least one non-3D among these sequences. 92 out of 99 patients had detectable edema. Mean GBM core volume was 42.8 cm^3^ (standard deviation = 29.2 cm^3^), while mean GBM edema volume was 52.6 cm^3^ (standard deviation 43.4 cm^3^). Table 1 summarizes clinical, surgical and prognostic variables for all patients included. 6 patients had missing data concerning the type of surgical operation that lead to GBM diagnosis and, among these, 5 had missing data regarding MGMT promoter methylation status, thus were not considered for survival analyses that included clinical-prognostic factors as regressors. The frequency maps of the distribution of the core and the edema are shown in Figure 1.

**Table 1:** Summary of patient characteristics and clinical/surgical variables.

Patient number	Age	Gender	Overall survival (months)	Radicality of resection	Stupp protocol	MGMT promoter methylation	ECOG performance status	Core volume (cm^3^)	Edema volume (cm^3^)
1	67	Male	29.74	PR	Yes	Yes	1	38.52	14.94
2	59	Male	12.37	PR	Yes	No	0	64.04	54.12
3	50	Male	10.03	PR	Yes	Yes	1	123.05	12.63
4	62	Male	10.95	PR	Yes	Yes	1	69.99	41.14
5	74	Male	3.65	PR	Yes	Yes	0	22.46	72.79
6	67	Male	4.21	PR	Yes	No	1	30.61	16.83
7	41	Male	8.98	GTR	Yes	No	0	22.07	45.92
8	77	Male	16.35	PR	Yes	Yes	2	74.36	74.75
9	47	Male	18.42	GTR	Yes	No	1	66.28	40.46
10	62	Male	10.16	GTR	Yes	No	1	11.69	0.00
11	61	Male	54.47	GTR	Yes	Yes	0	17.25	1.77
12	54	Male	19.18	GTR	Yes	No	1	57.16	0.00
13	69	Male	9.97	PR	Yes	No	1	54.36	4.88
14	61	Male	19.51	GTR	Yes	No	1	53.36	17.18
15	71	Male	26.71	PR	Yes	Yes	1	60.96	18.92
16	68	Female	1.81	PR	Yes	No	3	62.69	24.04
17	45	Male	4.8	PR	Yes	No	1	12.95	63.74
18	75	Male	2.63	B	No	No	3	34.12	80.16
19	64	Male	21.48	PR	Yes	Yes	1	35.04	10.97
20	65	Male	10.95	PR	Yes	No	2	45.30	90.41
21	71	Male	7.37	GTR	Yes	Yes	1	23.61	109.65
22	37	Male	16.97	PR	Yes	No	0	32.65	147.66
23	44	Female	3.55	PR	Yes	No	2	140.88	26.57
24	50	Male	30.1	PR	Yes	No	1	115.76	30.71
25	67	Male	2.2	PR	Yes	No	2	24.80	0.00
26	45	Male	47.63	GTR	Yes	Yes	0	9.75	0.00
27	54	Male	2.27	PR	No	No	3	40.24	83.20
28	41	Male	14.74	PR	Yes	Yes	0	49.89	40.21
29	76	Male	8.88	PR	Yes	Yes	2	27.50	92.32
30	60	Female	2.34	PR	Yes	Yes	2	68.30	92.09
31	75	Female	2.96	PR	Yes	No	2	82.85	139.36
32	67	Female	29.64	PR	Yes	No	0	33.12	62.50
33	65	Male	7.89	PR	Yes	Yes	2	82.75	74.66
34	69	Male	32.7	PR	Yes	Yes	2	53.33	5.99
35	65	Male	21.78	PR	Yes	No	0	50.12	96.74
36	54	Male	26.38	PR	No	Yes	0	11.08	67.66
37	76	Female	2.24	PR	Yes	Yes	3	62.96	89.49
38	53	Female	12.14	PR	Yes	Yes	0	17.48	57.65
39	72	Female	4.61	PR	Yes	Yes	3	101.71	35.15
40	75	Male	20.69	PR	Yes	Yes	1	34.14	24.00
41	73	Male	3.98	PR	Yes	No	3	7.66	74.58
42	71	Female	18.55	PR	Yes	Yes	1	38.93	35.35
43	68	Male	2.73	PR	No	No	1	53.32	9.71
44	64	Female	2.73	PR	No	Yes	3	10.32	7.67
45	79	Female	6.97	PR	Yes	No	2	26.50	46.87
46	70	Female	6.35	PR	Yes	No	2	16.23	89.81
47	63	Male	7.5	PR	Yes	No	1	21.86	62.65
48	61	Male	10.59	PR	Yes	No	0	88.58	60.82
49	57	Male	13.88	PR	Yes	No	0	31.00	19.34
50	67	Female	14.41	PR	Yes	No	0	22.91	132.54
51	67	Male	0.99	PR	Yes	Yes	2	92.60	112.73
52	67	Female	25.36	GTR	Yes	Yes	0	73.79	121.33
53	76	Male	3.72	B	No	Yes	2	46.32	19.87
54	45	Male	26.45	PR	Yes	No	0	5.54	17.79
55	70	Male	16.18	PR	Yes	No	1	59.95	99.98
56	56	Male	23.88	PR	Yes	Yes	0	22.27	10.18
57	73	Female	5.76	PR	No	Yes	2	76.76	7.81
58	56	Male	15.1	PR	Yes	No	1	44.63	115.24
59	73	Female	20.76	PR	Yes	Yes	2	84.88	15.82
60	68	Female	10.53	PR	Yes	No	0	11.89	14.22
61	54	Male	13.59	GTR	Yes	No	0	42.37	211.67
62	69	Female	37.24	GTR	Yes	Yes	1	15.29	37.05
63	68	Female	17.93	PR	Yes	No	1	25.22	107.82
64	70	Male	12	PR	Yes	Yes	1	29.16	25.96
65	64	Female	4.08	GTR	Yes	No	3	29.36	17.02
66	59	Male	15.66	PR	Yes	Yes	1	57.72	135.79
67	46	Male	31.61	PR	Yes	No	1	28.84	107.97
68	47	Male	2.99					13.94	2.13
69	67	Male	10.13	GTR	Yes	No	2	17.77	86.51
70	77	Female	2.99					16.66	0.00
71	81	Female	9.97	GTR	Yes	Yes	3	9.02	34.64
72	48	Male	14.97	PR	Yes	No	0	32.05	144.50
73	73	Female	1.68				4	85.39	50.88
74	45	Male	20.1	PR	Yes	No	2	18.44	35.37
75	70	Male	5.99	PR	Yes	Yes	1	16.33	47.73
76	53	Male	23.29	PR	Yes	Yes	1	15.00	52.24
77	58	Male	23.62	GTR	Yes	Yes	0	40.79	1.29
78	72	Male	21.19		Yes	No	2	41.74	38.76
79	71	Male	10.56	GTR	Yes	Yes	1	97.37	36.70
80	44	Male	3.72					60.30	0.00
81	65	Male	14.14	PR	Yes	No	1	23.56	11.66
82	78	Male	10.36	PR	Yes	No	1	31.48	59.71
83	71	Male	5.82				1	25.50	6.55
84	54	Male	13.06	PR	Yes	No	1	15.74	3.18
85	70	Male	6.35	PR	Yes	Yes	0	18.04	71.39
86	39	Male	22.4	GTR	Yes	No	1	103.34	39.18
87	71	Male	9.51	PR	Yes	No	0	48.60	67.92
88	74	Female	16.25	PR	Yes	Yes	1	100.66	94.33
89	65	Male	21.88	GTR	Yes	No	0	14.60	0.00
90	60	Female	23.45	GTR	Yes	No	0	22.01	50.52
91	64	Male	4.21	PR	Yes	No	2	49.57	50.95
92	68	Male	4.14	PR	Yes	Yes	1	0.68	44.18
93	20	Female	14.67	PR	No	No	0	27.00	47.81
94	57	Male	18.82	GTR	Yes	No	0	24.24	110.77
95	68	Male	24.31	PR	Yes	Yes	1	32.27	82.37
96	44	Male	13.59	PR	Yes	No	0	17.12	47.98
97	64	Female	21.71	PR	Yes	Yes	1	31.39	108.83
98	47	Female	25.26	GTR	Yes	Yes	1	30.28	100.25
99	66	Female	40.49	GTR	Yes	Yes	0	70.30	2.08

GTR, gross total resection; PR, partial resection; B, biopsy; blank cells represent missing data.

### 3.1. Overlap percentages: descriptive statistics and frequencies

**Figure 2:**
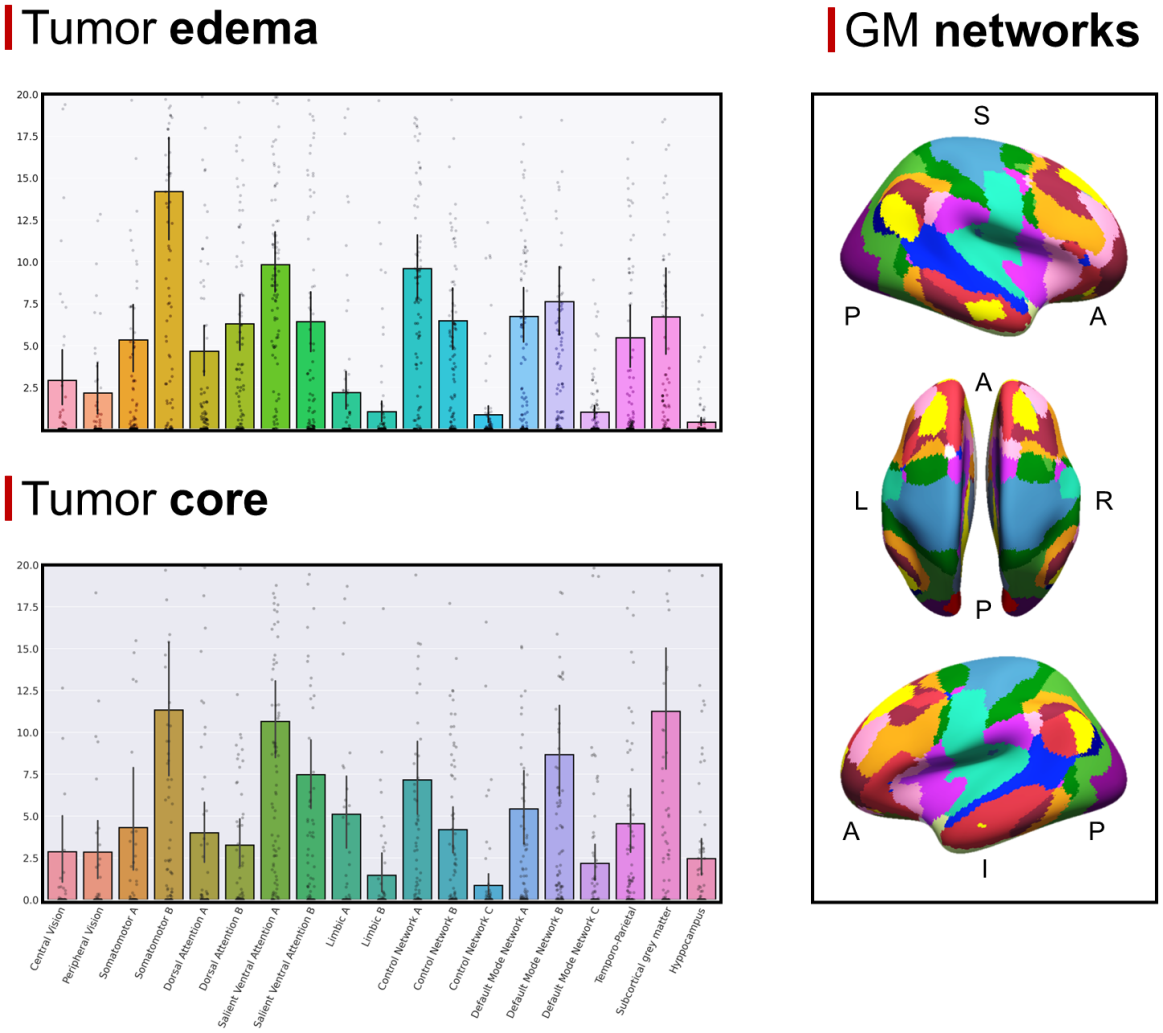
**(Top left section)** Mean overlap percentages between the GBM edema and Yeo’s GMNs, plus subcortical GM nuclei and hippocampi. **(Bottom left section)** Mean overlap percentages between the GBM core and Yeo’s GMNs, plus subcortical GM nuclei and hippocampi. **(Right section)** Yeo’s GMN atlas is depicted with colors representing different networks (subcortical gray matter nuclei and hippocampi are not shown).

**Figure 3:**
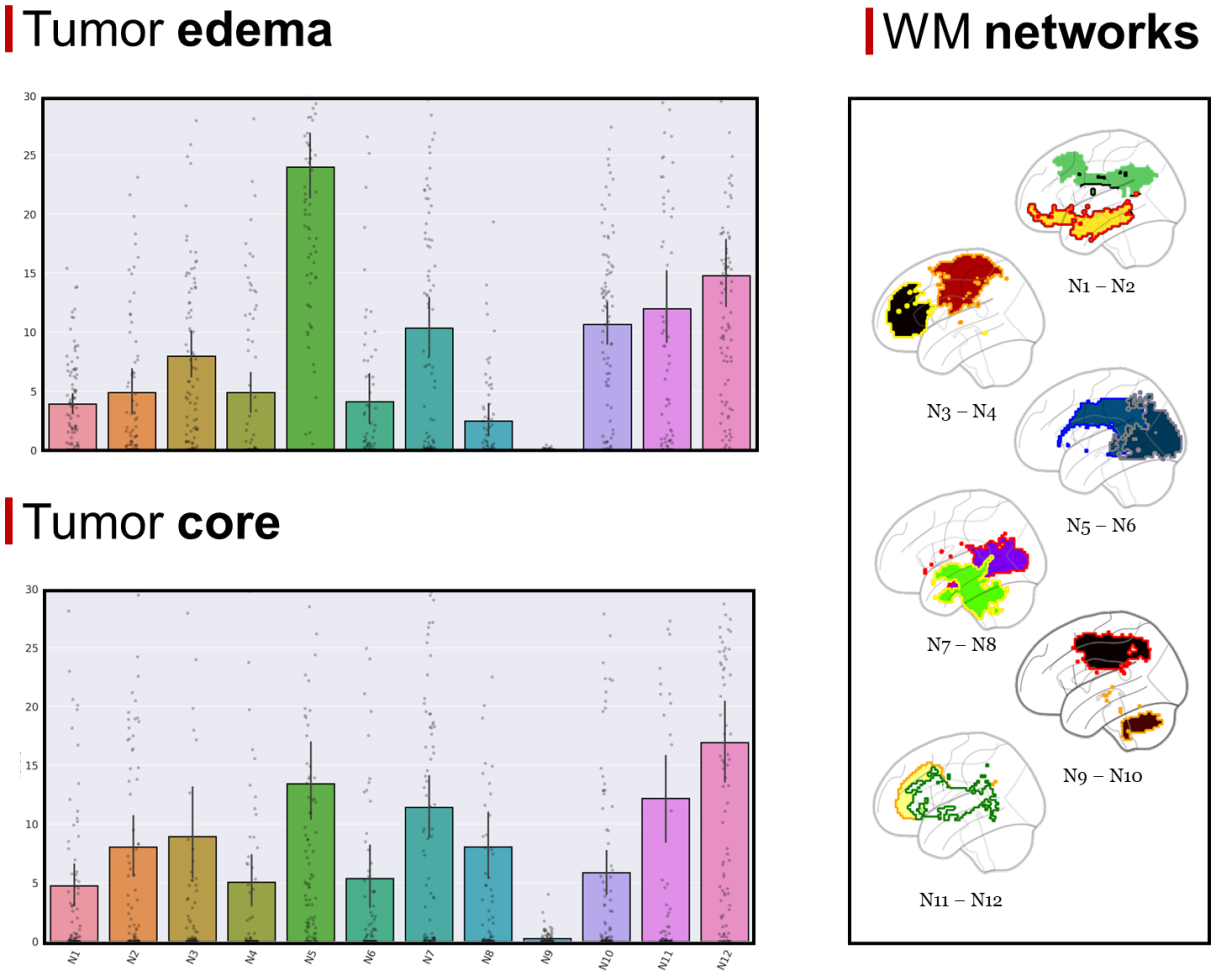
**(Top left section)** Mean overlap percentages between the GBM edema and Peer’s 12 WMNs. **(Bottom left section)** Mean overlap percentages between GBM core and Peer’s 12 WMNs. **(Right section)** Peer’s 12 WMNs are shown in sagittal view, with colors representing different networks: green for N1 (Cingulum and associated tracts), yellow with red border for N2 (Uncinate and middle temporal lobe tracts), red with orange border for N3 (Sensorimotor superficial white-matter system), black with yellow border for N4 (Forceps minor system), blue with dark blue border for N5 (superior longitudinal fasciculus system), blue with gray border for N6 (Visual superficial white-matter system), violet with red border for N7 (Inferior longitudinal fasciculus system), green with yellow border for N8 (Inferior corticospinal Tract), brown with orange border for N9 (Posterior cerebellar tracts), black with red border for N10 (Dorsal frontoparietal tracts), yellow with orange border for N11 (Deep frontal white matter), white with green border for N12 (Ventral frontoparietal tracts).

The GBM core mostly overlapped with the following GMNs: somatomotor B (mean overlap percentage = 11.3%), salient ventral attention A (10.6%), default mode network B (8.6%), salient ventral attention B (7.4%), control network A (7.1%), limbic A (5.1%). Regarding WMNs, the most involved ones were: N12 (ventral frontoparietal tracts, 16.9%), N5 (superior longitudinal fasciculus system, 13.4%), N11 (deep frontal WM, 12.1%), N7 (inferior longitudinal fasciculus system, 11.4%). Concerning edema, the most overlapped GMNs were: somatomotor B (14.2%), salient ventral attention A (9.8%), control network A (9.6%), default mode network B (7.6%), default mode network A (6.7%). As for WMNs: N5 (superior longitudinal fasciculus system, 24%), N12 (ventral frontoparietal tracts, 14.8%), N11 (deep frontal WM 12%). Overlaps with the aforementioned networks altogether accounted for 50% of overlap within each of the four overlap categories. Descriptive statistics for computed overlap percentages are shown in Tables 2, 3, while mean overlap percentages are represented graphically in Figures 2, 3.

**Table 2:** Descriptive statistics of overlap percentages between the GBM core and functional brain networks.

Functional networks		CORE		
		Involvement frequency (threshold 0.5%)^*^	Mean	Standard deviation
Gray matter networks	Central Vision	16.16%	2.9%	10.5%
	Peripheral Vision	18.18%	2.8%	8.9%
	Somatomotor A	22.22%	4.3%	15.0%
	Somatomotor B	54.55%	11.3%	20.3%
	Dorsal Attention A	26.26%	4.0%	9.5%
	Dorsal Attention B	32.32%	3.3%	7.4%
	Salient Ventral Attention A	78.79%	10.6%	11.4%
	Salient Ventral Attention B	57.58%	7.5%	10.5%
	Limbic A	30.30%	5.1%	10.9%
	Limbic B	15.15%	1.5%	6.1%
	Control network A	54.55%	7.2%	11.2%
	Control network B	46.46%	4.2%	6.8%
	Control network C	11.11%	0.9%	3.2%
	Default Mode Network A	47.47%	5.4%	11.2%
	Default Mode Network B	68.69%	8.7%	13.9%
	Default Mode Network C	29.29%	2.2%	5.5%
	Temporo-Parietal	49.49%	4.5%	9.4%
	Subcortical gray matter (basal ganglia and thalami)	55.56%	11.2%	18.6%
	Hippocampus	35.35%	2.5%	5.3%
White matter networks	N1 (Cingulum and associated tracts)	44.44%	4.7%	8.9%
	N2 (Uncinate and middle temporal lobe tracts)	52.53%	8.0%	13.3%
	N3 (Sensorimotor superficial white-matter system)	40.40%	8.9%	19.2%
	N4 (Forceps minor system)	32.32%	5.0%	10.7%
	N5 (Superior longitudinal fasciculus system)	83.84%	13.4%	16.0%
	N6 (Visual superficial white-matter system)	40.40%	5.3%	13.2%
	N7 (Inferior longitudinal fasciculus system)	59.60%	11.4%	12.9%
	N8 (Inferior corticospinal Tract)	43.43%	8.0%	14.6%
	N9 (Posterior cerebellar tracts)	18.18%	0.2%	0.6%
	N10 (Dorsal frontoparietal tracts)	46.46%	5.8%	9.6%
	N11 (Deep frontal white matter)	46.46%	12.2%	18.8%
	N12 (Ventral frontoparietal tracts)	82.83%	16.9%	16.6%

*overlap percentages < 0.5% were not counted.

**Table 3:** Descriptive statistics of overlap percentages between the GBM edema and functional brain networks.

Functional networks		EDEMA		
		Involvement frequency (threshold 0.5%)	Mean	Standard deviation %
Gray matter networks	Central Vision	22.83%	2.92%	8.46%
	Peripheral Vision	25.00%	2.16%	7.66%
	Somatomotor A	50.00%	5.33%	10.12%
	Somatomotor B	80.43%	14.21%	14.53%
	Dorsal Attention A	54.35%	4.66%	7.57%
	Dorsal Attention B	68.48%	6.30%	7.94%
	Salient Ventral Attention A	89.13%	9.83%	8.59%
	Salient Ventral Attention B	58.70%	6.41%	8.54%
	Limbic A	27.17%	2.19%	5.54%
	Limbic B	17.39%	1.06%	3.13%
	Control network A	83.70%	9.59%	9.08%
	Control network B	71.74%	6.48%	8.90%
	Control network C	19.57%	0.86%	2.44%
	Default Mode Network A	70.65%	6.73%	8.34%
	Default Mode Network B	76.09%	7.62%	10.07%
	Default Mode Network C	35.87%	1.02%	1.88%
	Temporo-Parietal	52.17%	5.47%	9.20%
	Subcortical gray matter (basal ganglia and thalami)	65.22%	6.72%	12.93%
	Hippocampus	18.48%	0.43%	1.14%
White matter networks	N1 (Cingulum and associated tracts)	73.91%	3.92%	3.84%
	N2 (Uncinate and middle temporal lobe tracts)	53.26%	4.90%	8.82%
	N3 (Sensorimotor superficial white-matter system)	77.17%	7.96%	9.56%
	N4 (Forceps minor system)	39.13%	4.88%	8.47%
	N5 (Superior longitudinal fasciculus system)	94.57%	23.96%	13.24%
	N6 (Visual superficial white-matter system)	43.48%	4.09%	10.07%
	N7 (Inferior longitudinal fasciculus system)	65.22%	10.34%	11.71%
	N8 (Inferior corticospinal Tract)	38.04%	2.47%	6.63%
	N9 (Posterior cerebellar tracts)	0%	0.03%	0.07%
	N10 (Dorsal frontoparietal tracts)	81.52%	10.67%	8.61%
	N11 (Deep frontal white matter)	67.39%	11.98%	14.78%
	N12 (Ventral frontoparietal tracts)	93.48%	14.80%	14.28%

Only the 92 patients with edema were included in these calculations.

*overlap percentages < 0.5% were not counted.

### 3.2. Correlation analyses between overlap percentages

We found several significant positive correlations between overlap percentages, some of which regarded functionally inter-related GMNs and WMNs (23): for both core and edema, overlap with N2 (uncinate and middle-temporal lobe tracts) positively correlated with default mode network B overlap (core: *R* = 0.62, *p* < 0.0001; edema; *R* = 0.58, *p* < 0.0001), the same was found between N6 (visual superficial WM system), central (core: *R* = 0.9, *p* < 0.0001; edema: *R* = 0.95, *p* < 0.0001) and peripheral vision networks (core: *R* = 0.55, *p* < 0.0001; edema: *R* = 0.6, *p* < 0.0001), as well as between N3 (sensorimotor superficial WM system) and somatomotor A network (core: *R* = 0.71, *p* < 0.0001; edema: *R* = 0.8, *p* < 0.0001). Only for core, overlap with N1 positively correlated with control network C (*R* = 0.49, *p* < 0.0001) and default mode network A (*R* = 0.37, *p* = 0.0001). Only for edema, overlap with N12 positively correlated with somatomotor B (R = 0.71, *p* < 0.0001). The complete results of correlation analyses are shown in Supplementary Figures S1, S2 (only results that survived Bonferroni correction are shown), Concerning correlations with the radicality of surgery, we found that overlaps between the GBM core and somatomotor-B (*R* = 0.23, *p* = 0.03), between the GBM core and DMN-B (R = 0.24, *p* = 0.02) and between the edema and somatomotor-B (R = 0.24, *p* = 0.02) were associated to a wider resection, however, these correlations did not survive Bonferroni correction for multiple comparisons (the complete set of results are shown in Supplementary Table S4).

### 3.3. Canonical correlation analysis

**Figure 4:**
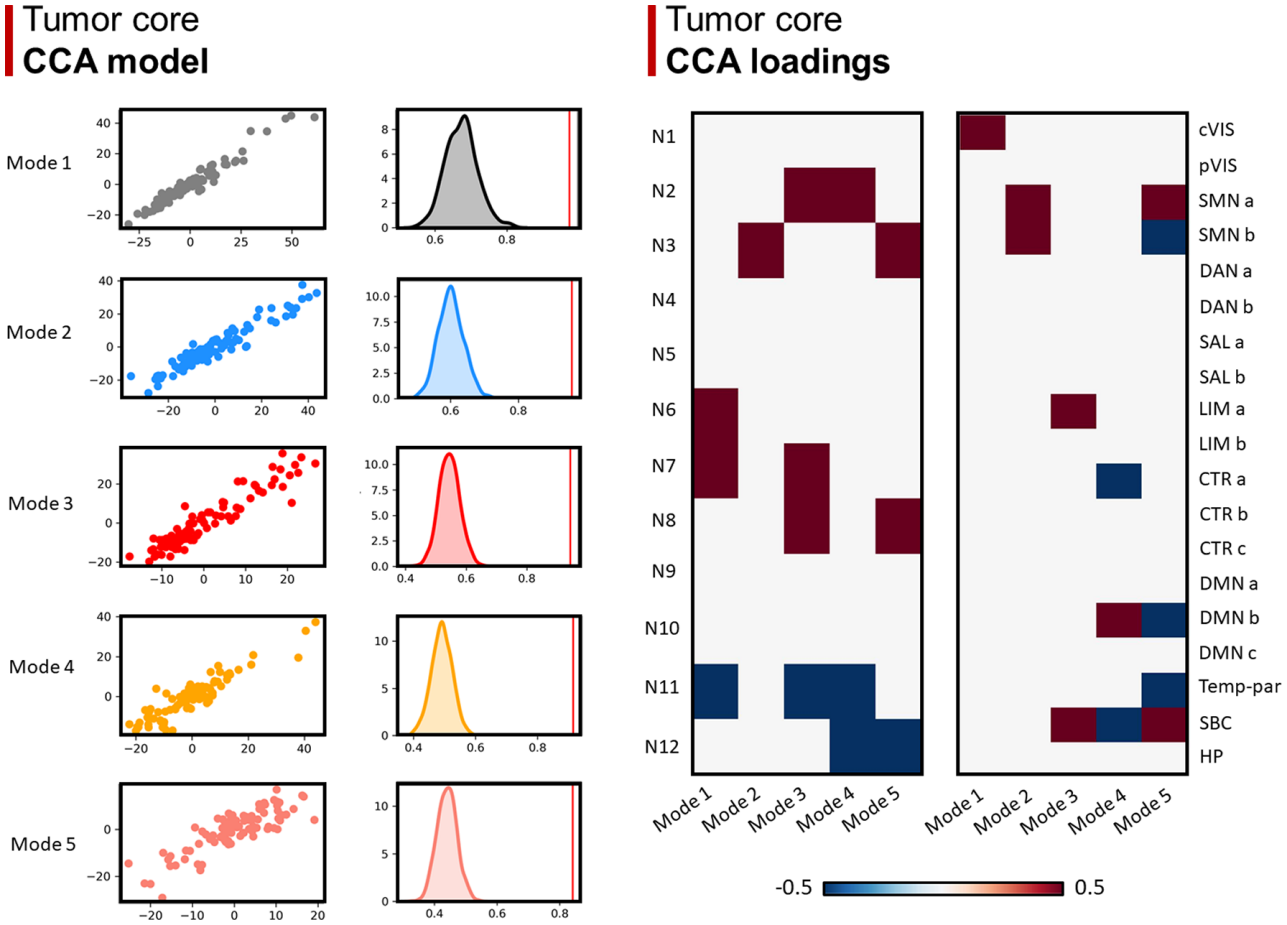
Canonical correlation analysis (CCA) between WMN and GMN overlap percentages with GBM core. **Left panel (CCA model)**: 5 modes were identified (*r* > 0.8; *p* < 0.001), surviving statistical significance after a permutation (*n* = 1,000) comparison approach (*p* < 0.0001). **Right panel (CCA loadings)**: patterns (or modes) of WMN and GMN overlap with the GBM core are shown for each mode. N1 to N12 represent the white matter functional networks (WMNs); cVIS: central vision; pVIS: peripheral vision; SMN a: somatomotor A; SMN b: somatomotor B; DAN a: dorsal attention A; DAN b: dorsal attention B; SAL a: salient ventral attention A; SAL b: salient ventral attention B; LIM a: limbic A; LIM b: limbic B; CTR a: control A; CTR b: control B; CTR C: control C; DMN a: default-mode A; DMN b: default-mode B; DMN c: default-mode C; Temp-par: temporo-parietal; SBC: subcortical gray matter nuclei; HP: hippocampus.

Concerning the GBM core, 5 modes were identified as statistically significant compared to a random distribution (*n*=1000; *p* < 0.05; Figure 4). The first mode highlighted a relationship mainly involving visual superficial WM system (N6) and inferior longitudinal fasciculus system (N7) from the WM side, and the central vision network from the GM matrix; the second mode was mainly related to sensorimotor superficial WM system (N3), as well as the somatomotor A and B GMNs; mode 3 was related to uncinate and middle temporal lobe tracts (N2), inferior longitudinal fasciculus system (N7) and limbic A network; mode 4 to N2 and default-mode network B; mode 5 to N3, inferior corticospinal tract (N8), somatomotor A and subcortical GM nuclei. Regarding negative loadings: deep frontal WM (N11) was negatively related to modes 1, 3 and 4, while ventral frontoparietal tracts (N12) was to mode 4 and 5. Moreover, mode 4 was negatively related to control network A and subcortical GM nuclei, whereas mode 5 was to somatomotor B, default-mode network B and temporo-parietal network.

**Figure 5:**
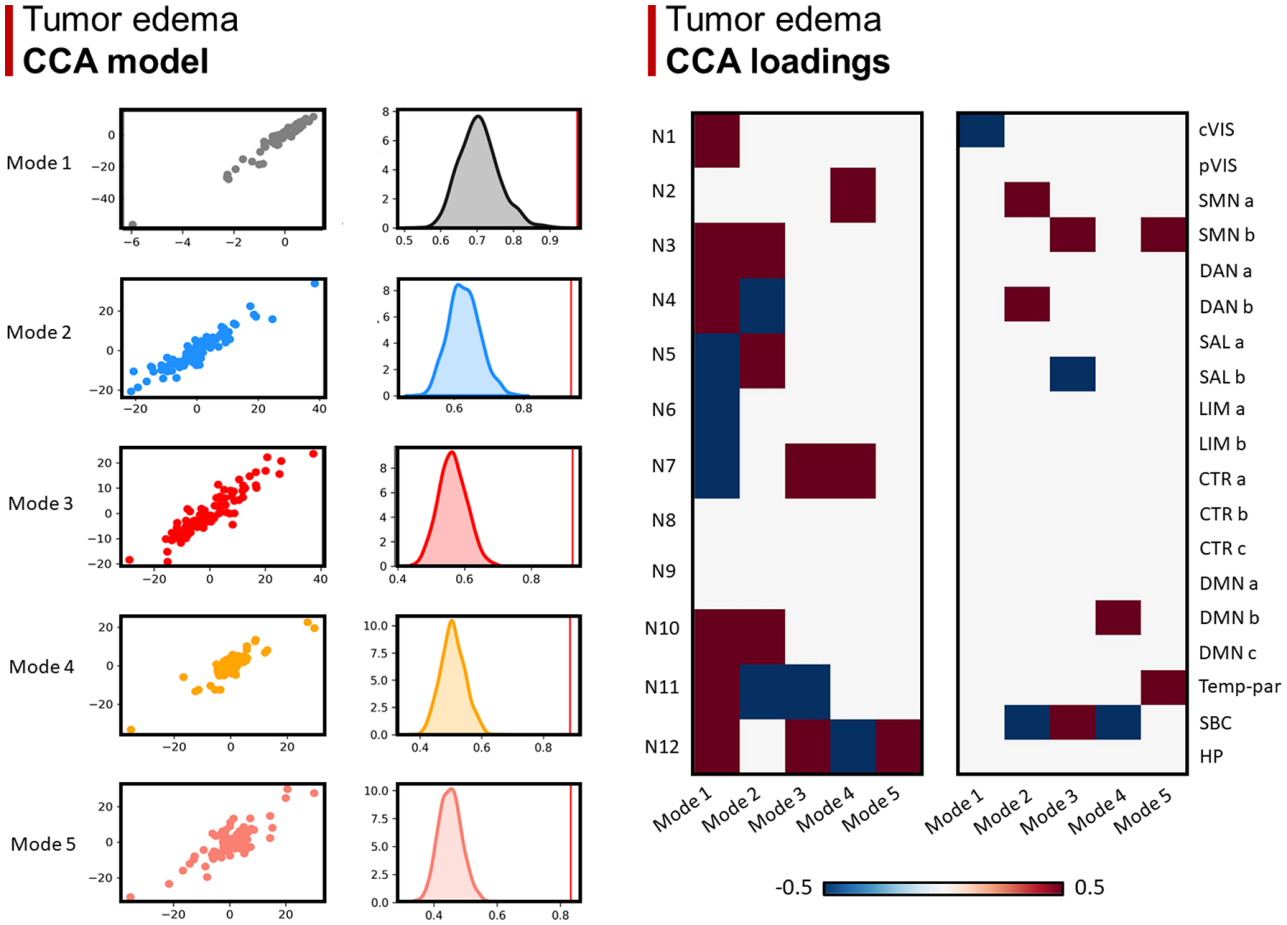
Canonical correlation analysis (CCA) between WMN and GMN overlap percentages with GBM edema. **Left panel (CCA model)**: 5 modes were identified (*r* > 0.8; *p* < 0.001), surviving statistical significance after a permutation (*n* = 1,000) comparison approach (*p* < 0.0001). **Right panel (CCA loadings)**: patterns (or modes) of WMN and GMN overlap with the GBM edema are shown for each mode. N1 to N12 represent the white matter functional networks (WMNs); cVIS: central vision; pVIS: peripheral vision; SMN a: somatomotor A; SMN b: somatomotor B; DAN a: dorsal attention A; DAN b: dorsal attention B; SAL a: salient ventral attention A; SAL b: salient ventral attention B; LIM a: limbic A; LIM b: limbic B; CTR a: control A; CTR b: control B; CTR C: control C; DMN a: default-mode A; DMN b: default-mode B; DMN c: default-mode C; Temp-par: temporo-parietal; SBC: subcortical gray matter nuclei; HP: hippocampus.

Lastly, 5 modes were identified for network overlaps with the GBM edema, in which the number of positive and negative relationships decrease from mode 1 to 5 (Figure 5).

### 3.4. ANOVA

Four ANOVA tests showed significant differences in the degree of overlap between GBM lesions and different brain networks. In particular, this was true for overlaps between the GBM core and GMNs (*F* = 7.8, *p* < 0.001), the GBM edema and GMNs (*F* = 17.5, *p* < 0.001), the GBM core and WMNs (*F* = 10.9, *p* < 0.001), as well as between the GBM edema and WMNs (*F* = 39.3, *p* < 0.001). Significant Bonferroni-corrected post-hoc comparisons are shown in Supplementary Tables S1, S2.

### 3.5. Comparison between the GBM core and edema overlap percentages

The mean percentage overlap with the hippocampi was significantly lower for the edema region (0.4%) as compared with the lesion core (2.5%) (*p* <0.001). Concerning WMNs, N5 (superior longitudinal fasciculus system) and N10 (dorsal fronto-parietal tracts) overlapped significantly more with the edema region (24 and 10.7%, respectively) than the core region (13.4 and 5.8%, respectively) (both *p*-values<0.001). In contrast, N8 and N9 overlapped more with the core (8 and 0.2%, respectively) than with the edema region (2.5 and 0.03%, respectively) (p-values = 0.004 and 0.001, respectively). Other comparisons did not reach significance after Bonferroni correction for multiple comparisons.

### 3.6. Association between OS and GBM distribution across functional networks

Multiple linear regression models are summarized in Table 4. The models only including clinical-prognostic variables and core size were statistically significant with an explained variance (adjusted *R*^2^) of 0.34 (*F* = 8.7, *p* < 0.001); significant regressors were ECOG with *β* = −4 and *p* < 0.001, radicality of surgery with *β* = 2.5 and *p* = 0.01, MGMT status with *β* = 5.8 and *p* = 0.003. Similar results were found for the model including edema size with an explained variance (adjusted *R*^2^) of 0.31 (*F* = 7.5, *p* < 0.001), significant regressors were ECOG with *β* = −4 and *p* < 0.001, radicality of surgery with *β* = 2.2 and *p* = 0.02, MGMT status with *β* = 4.2 and *p* = 0.029.

**Table 4:** Multiple linear regression models (dependent variable is overall survival/OS for all models).

CORE					EDEMA				
Model: Independent variables or regressors: GBM core volume; age; ECOG performance status; radicality of surgical resection (biopsy, partial resection, gross total resection); MGMT promoter methylation status; Stupp protocol.					Model: Independent variables or regressors: GBM edema volume; age; ECOG PS; radicality of surgical resection (biopsy, partial resection, gross total resection); MGMT promoter methylation status; Stupp protocol.				
Model parameters	adjusted *R*^2^	0.34			Model parameters	adjusted *R*^2^	0.31		
	*F*	8.7				*F*	7.5		
	*p*	**<0.001**				*p*	**<0.001**		
Significant regressors	Variable	MGMT	ECOG	Radicality of surgery	Significant regressors	Variable	MGMT	ECOG	Radicality of surgery
	*β*	5.8	−4	2.5		*β*	4.2	-4	2.2
	*p*	0.003	<0.001	0.01		*p*	0.029	<0.001	0.02
Model: Independent variables or regressors: percentages of overlap between GBM core and GMNs; GBM core volume; age; ECOG performance status; radicality of surgical resection (biopsy, partial resection, gross total resection); MGMT promoter methylation status; Stupp protocol.					Model: Independent variables or regressors: percentages of overlap between GBM edema and GMNs; GBM edema volume; age; ECOG performance status; radicality of surgical resection (biopsy, partial resection, gross total resection); MGMT promoter methylation status; Stupp protocol.				
Model parameters	adjusted *R*^2^	0.2			Model parameters	adjusted R^2^	0.19		
	*F*	2				*F*	1.8		
	*p*	**0.01**				*p*	**0.025**		
Significant regressors	Variable	MGMT	ECOG	Radicality of surgery	Significant regressors	Variable	ECOG		
	*β*	7.2	−3.9	2.5		*β*	−4.2		
	*p*	0.005	0.006	0.04		*p*	0.002		
Model: Independent variables or regressors: percentages of overlap between GBM core and WMNs; GBM core volume; age; ECOG PS; radicality of surgical resection (biopsy, partial resection, gross total resection); MGMT promoter methylation status; Stupp protocol.					Model: Independent variables or regressors: percentages of overlap between GBM edema and WMNs; GBM edema volume; age; ECOG performance status; radicality of surgical resection (biopsy, partial resection, gross total resection); MGMT promoter methylation status; Stupp protocol.				
Model parameters	adjusted *R*^2^	0.32			Model parameters	adjusted *R*^2^	0.33		
	*F*	3.6				*F*	3.5		
	*p*	**<0.001**				*p*	**<0.001**		
Significant regressors	Variable	MGMT	ECOG	N12	Significant regressors	Variable	ECOG	Radicality of surgery	
	*β*	6.3	−3.7	3.6		*β*	−3.4	2.2	
	*p*	0.003	0.004	0.03		*p*	0.004	0.03	

After adding network overlap percentages to the clinical variables as regressors, we did not obtain an increase in explained OS variance. In particular, when we included the core-GMNs overlap percentages, adjusted *R*^2^ decreased to 0.20 (*F* = 2, *p* = 0.01), significant regressors were ECOG with *β* = −3.9 and *p* = 0.006, radicality of surgery with β = 2.5 and *p* = 0.04, MGMT status with *β* = 7.2 and *p* = 0.005. Similarly, when we included edema-GMNs overlap percentages as regressors, the explained variance (adjusted *R*^2^) further decreased to 0.19 (*F* = 1.8, *p* = 0.025), significant regressors were ECOG with *β* = −4.2 and *p* = 0.002, while radicality of surgery and MGMT status’ value of ps were 0.069 and 0.064, respectively. When core-WMN overlap percentages were added as regressors, the explained variance (adjusted R^2^) was 0.32 (*F* = 3.6, *p* < 0.001), significant regressors were ECOG with *β* = −3.7 and *p* = 0.004, MGMT status with *β* = 6.3 and *p* = 0.003, N12 with *β* = 3.6 and *p* = 0.03, while the value of p for radicality of surgery was 0.063. Finally, when we included edema-WMN overlap percentages as regressors, the adjusted R^2^ was 0.33 (*F* = 3.5, *p* < 0.001), significant regressors were ECOG with *β* = −3.4 and *p* = 0.004, radicality of surgery with *β* = 2.2 and *p* = 0.03, while the value of p for MGMT status was 0.056.

Models only including network overlap percentages as regressors, as well as regression models including “not normalized” and “alternative” overlap percentages (see “Tumor-networks overlap computation” paragraph in the *Methods* section), are shown in Supplementary Table S3.

**Figure 6:**
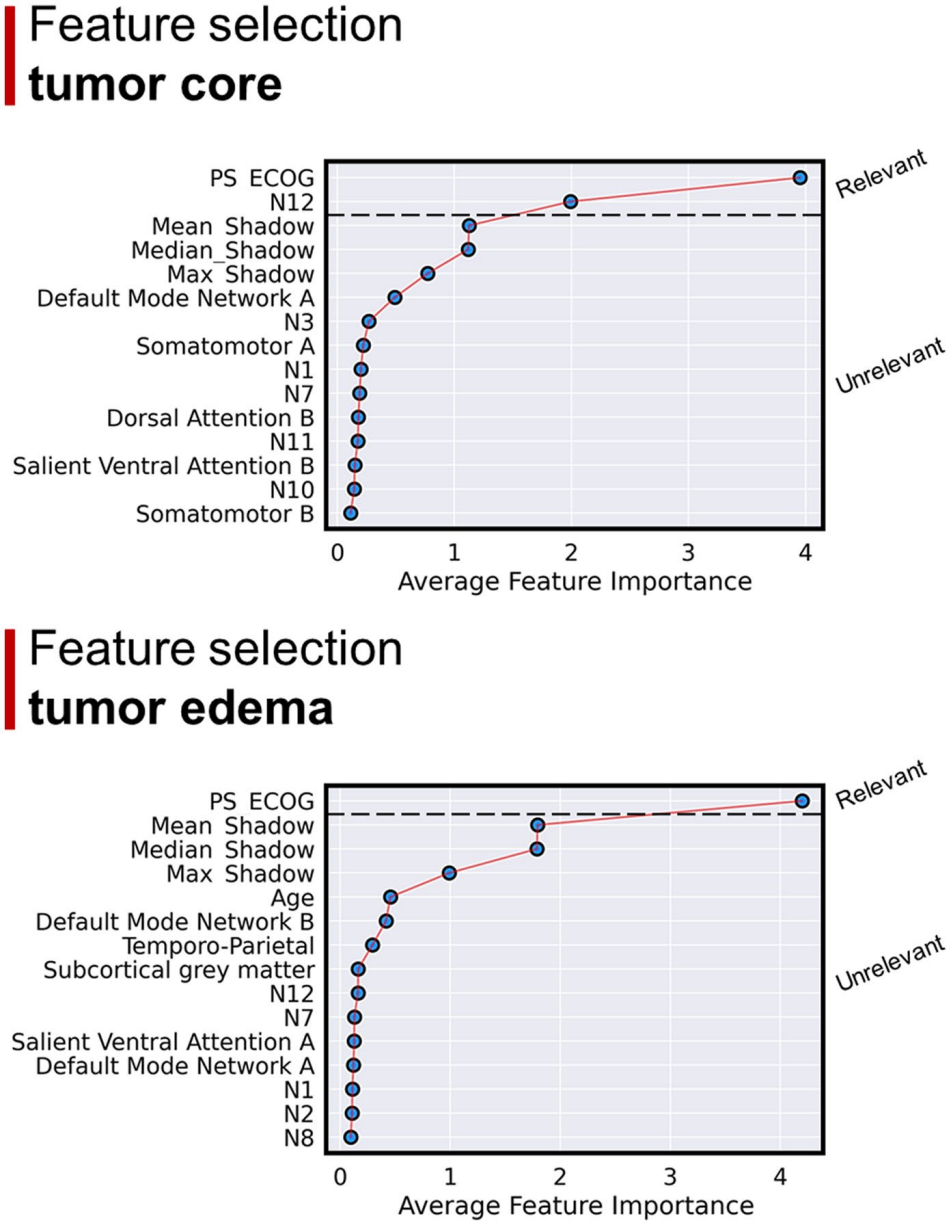
Results of the Boruta regression analysis. Considering GBM core overlap percentages: the main regressors explaining OS were ECOG and overlap with N12. Considering GBM edema overlap percentages: ECOG was the only relevant feature for OS regression.

According to the non-linear approach (i.e., Boruta analysis), the most important features for OS were ECOG and overlap between tumor core with N12. When overlaps with edema were considered, ECOG was the only relevant feature for OS (Figure 6).

## 4. Discussion

### 4.1. Main results

Our data shows that GBMs are distributed differently across GMNs and WMNs: the GBM preferentially locates in associative networks, confirming the results that Mandal et al. obtained in a different GM networks parcellation (13). In particular, we found five main patterns of GBM core distribution across functional networks. Furthermore, although we found similar values of mean edema-network overlap percentages, edema does not seem to have a well-defined network-based anatomical distribution. The second main result is that OS was not clearly associated with the distribution of GBM across functional brain networks. In contrast to previous similar studies, we also considered the functional network anatomy of WM, hence highlighting its potential importance, as GBM is predominantly a WM disease.

### 4.2. GBM distribution across functional brain networks

Concerning GM and core lesions, about 14% of the overlap is with subcortical nuclei (thalami and basal ganglia); about 50% of the overlap is with a small number of GMNs – six networks – of which five are associative networks. The only sensory-motor network is the somatomotor B network, which spans the ventral part of the posterior frontal and anterior parietal lobes. The other five include fronto-parietal networks both on the lateral and medial surfaces of the brain. Naturally, the edema region involves more overlap (~93%) with GMNs compared to subcortical GM nuclei, but also in this case around 50% of the overlap occurs with only five networks, of which four are associative in nature.

Regarding WMNs, about half of the region of the GBM core or edema overlaps with association WMNs connecting long-range fronto-temporo-parietal regions either ventrally (ventral frontoparietal tracts, deep frontal WM) or dorsally (superior longitudinal fasciculus system). More focal short-range tracts like the sensory-motor, dorsal or uncinate tracts overlapped less with core or edema regions. Another interesting finding was the lower involvement of the hippocampi by the edema region, compared with the core. This is explained since edema typically distributes within WM, with relative sparing of GM (36).

Remarkably, canonical correlation analysis allowed us to identify five main patterns in which GBM core distributes across WMNs and GMNs. As far as the GBM core is concerned, the first pattern mainly implies visual GMNs and WMNs, the second somatomotor GMNs and WMNs, while the third one mainly regarded temporal networks (uncinate and middle temporal lobe tracts/N2, inferior longitudinal fasciculus system/N7 and limbic network). The fourth mode concerns uncinate and middle temporal lobe tracts (N2) and default-mode network B; the fifth pattern, instead, reflects the course of the whole corticospinal tract, from the cortex, passing through the internal capsule and adjacent basal ganglia, to the inferior portion of the pathway. Interestingly, such pattern only regards the dorsal part of the somatomotor network, while it is negatively correlated with the ventral portion. These findings might indicate that the potential migration of GBM cells along the corticospinal tract preferentially occurs from/towards dorsal regions, for reasons yet to be clarified. Frontoparietal or deep frontal WM networks were negatively associated with these patterns, except for the second one. In particular, our data suggest that when GBMs have certain distribution modes, they tend not to affect specific networks: deep frontal WM (patterns 1, 3, 4), ventral frontoparietal tracts (patterns 4, 5), control network A (pattern 4), subcortical GM nuclei (pattern 4), somatomotor B (pattern 4), default-mode network B (pattern 5) or temporo-parietal network (pattern 5).

Concerning edema, the five identified modes were less classifiable, indicating a more diffuse involvement of networks, often extending far beyond the anatomical location of the GBM core. The great contrast in the number of loadings between different modes perhaps indicates the higher interindividual variability of edema extension, compared to the core: while GBM distribution seems to follow an anatomical (network-based) pattern, the edema follows an anatomical (not network-based) path involving the WM structure across many networks. Overall, the differences between the results found for the GBM edema and core, discussed above, suggest that the anatomical location of the latter one can be more effectively subdivided into a discrete number of categories or “patterns,” based on both GMNs and WMNs. On the contrary, the distribution of edema is characterized by a higher extent of variability and unpredictability.

### 4.3. Interrelated GMNs and WMNs are co-lesioned by GBM

It is well-known that glioma cells form synapses with neurons, in which a signaling based upon glutamate and other molecules generates self-amplifying auto/paracrine loops that are thought to contribute to several neoplastic processes including tumor growth and migration (44–48). Such molecules are neuron activity-dependent, thus recent studies have postulated a possible link between the aforementioned phenomena and neuronal activation itself (49–53). Moreover, Mandal et al. (25) used independent component analysis to decompose low- and high-grade glioma lesions into 3 principal areas of co-lesioned brain regions (“lesion covariance networks” or “LCNs”), which showed anatomical correspondence to different structural WM tracts and functional connectivity networks (obtained from Miller et al. (24)), interpreted as the tendency of glioma cells to migrate along neuronal networks that support glioma cell proliferation. In our study, we too obtained some evidence furtherly indicating a possible link between GBM localization or spreading and brain functional connectivity: correlation analyses showed that some functionally and anatomically inter-related GMNs and WMNs tended to be co-lesioned by GBM. In particular, Peer et al. (23) showed that N2 (uncinate and middle-temporal lobe tracts) has a high degree of functional correlation with the default mode network and, in our work, their involvements by GBM positively correlate one with another. Similarly, overlap with peripheral and central vision networks positively correlates with the associated WMN N6 (visual superficial WM system), as did somatomotor network A with N3 (sensorimotor superficial WM system). Such results practically mirror the “modes” of the GBM core distributions, identified through canonical correlation analysis. Additional remarkable results, coherent with Peer’s aforementioned inter-network correlations, were found between control network and N1 (cingulum and associated tracts), default mode network and N1, as well as between somatomotor B and N12 (ventral frontoparietal tracts), although the latter association was only found within perilesional edema. In Peer’s original article, among the superficial WMNs, N1, N2 and N12 strongly correlated with widely distributed GMNs (DMN, dorsal attention, ventral attention and frontoparietal control networks), suggesting a role for these networks in allowing communication between distant regions of GMNs. Conversely, other superficial networks mostly showed a high correlation with the overlying GMNs, possibly indicating the presence of short-range connections between them. Therefore, while the co-localisation of GBMs in N3 and somatomotor or N6 and visual networks might be predominantly explained by the shared topology of the networks, the joint involvement of N1 or N2 and the aforementioned associative GMNs might imply a contribution of their intrinsic activity. Hence, in line with the aforementioned studies and our results, we too hypothesize that the preferential distribution of GBM across certain functional networks might not merely reflect their most frequent anatomical locations, but may be, at least in part, influenced by the activity of brain functional networks themselves.

### 4.4. OS and GBM distribution across functional brain networks

According to a large amount of previous literature, we found that OS was predicted by MGMT methylation status, Surgery extension and ECOG performance status. The level of explained variance ranged from 31 to 34%. When we added GMN overlap percentages the variance explained (adjusted R^2^) was lower (around 20%), suggesting that this anatomical information does not help in predicting OS, while it was similar to the models only including clinical-prognostic variables when we added WMN overlap percentages. In line with linear regression results, Boruta regression showed that none of the GMN overlap percentages are important features for OS regression, while the extent to which the GBM core overlaps ventral frontoparietal tracts (N12) seems to have some role in predicting a longer survival. Around half of this network’s volume is constituted by components of well-defined anatomical tracts, in particular the anterior thalamic radiation, the superior longitudinal fasciculus and, to a lesser extent, the corticospinal tract. From a functional point of view, N12 is highly correlated to somatomotor, ventral and dorsal attention networks.

Overall, however, we did not find any robust association between OS and GBM distribution across functional brain networks. Also Mandal et al.’s (25) work was coherent with our findings, as the differences in OS that they found between LCNs were mainly driven by molecular determinants, rather than glioma distribution, and lost significance when distinguishing GBM from low-grade gliomas. In contrast, previously mentioned studies found that fMRI-derived data, in particular functional connectivity between intra-tumoral and extra-tumoral regions, were significantly associated with patient survival (27–29). Our interpretation of such discrepancy is that the mere overlap between GBM and brain networks is not informative enough of the actual functional impairment caused. Thus, we believe that *in-vivo* functional connectivity techniques (including either fMRI and/or neurophysiology) are needed to measure the real impact that the GBM-driven network dysfunction has on patient survival. Nonetheless, our work showed that the WM and its functional activity might harbor valuable information for a better understanding of GBM pathophysiology and its prognostication. In fact, other approaches to investigate the relationships between GBM and WM organization have already been tested: in their recently submitted work, Salvalaggio et al. discovered a novel negative prognostic factor, the density index (i.e., the density of WM fibers overlapped by GBMs), showing the promising potential of structural connectivity-based studies in neuro-oncology (54).

### 4.5. Limitations

This study has some limitations. (1) Yeo’s atlas is the most widely used for GMNs, although we cannot exclude that using different atlas would influence the results; (2) the lower age of the healthy subjects’ cohorts used for Yeo’s and Peer’s atlases compared to our patient sample; (3) the exact nature of the fMRI signal within WM, from which WMNs were identified, is still partially uncertain. While there is evidence that both task-evoked and resting state BOLD signals in WM seem to be, at least in part, caused by hemodynamic changes associated with neural WM activity, the existence of other sources of fMRI signals have been postulated, such as spiking-related metabolic demands and activity of astrocytes and NO-producing neurons (52). To date, the entire biophysical basis of fMRI signals within WM is not utterly understood (22). (4) Another limitation of the present study is the absence of a comparison of the topological network overlap data to functional connectivity modifications induced by GBM, investigable with fMRI. (5) Lastly, our work lacks neuropsychological data, which could be integrated in future studies.

### 4.6. Conclusion

In conclusion, the GBM core and edema preferentially overlap certain GMNs, specifically associative networks, and related WMNs, involved in cognitive functions. Five main patterns of GBM core distribution across functional networks were found. GBM lesions tended to impact jointly some interrelated white and gray matter functional systems, suggesting that tumor growth and spreading might not be independent of brain activity. Although the involvement of ventral frontoparietal tracts (N12) seems to have some role in predicting a longer survival, network-topology information is overall scarcely informative about OS.

## Data availability statement

The raw imaging data supporting the conclusions of this article will be made available by the authors, upon reasonable request.

## Ethics statement

The study was approved by the Ethical Committee of the Province of Padua (Comitato Etico per la Sperimentazione Clinica della Provincia di Padova n. 70n/AO/20). Written informed consent for participation was not required for this study in accordance with the national legislation and the institutional requirements.

## Author contributions

GS, LP, AS, and MC designed the study. VB, FV, FC, DD’A, MP, LD, VZ, and GL were in charge of the patients and provided clinical and survival data. GS, MG, MA, and AS generated the lesion masks. LP, GS, and AS analyzed the imaging data. GS, LP, AS, and MC participated in the interpretation of data. GS, AS, LP, and MC wrote the manuscript. All authors contributed to the article and approved the submitted version.

## Funding

MC was supported by Fondazione Cassa di Risparmio di Padova e Rovigo (CARIPARO)—Ricerca Scientifica di Eccellenza 2018 (Grant Agreement number 55403); Italian Ministero della Salute, Brain connectivity measured with high-density electroencephalography: a novel neurodiagnostic tool for stroke (NEUROCONN; RF-2018-1236689); Celeghin Foundation Padova (CUP C94I20000420007); BIAL foundation grant (No. 361/18); Horizon 2020 European School of Network Neuroscience—European School of Network Neuroscience (euSNN), H2020-SC5-2019-2 (Grant Agreement number 860563); Horizon 2020 research and innovation program; Visionary Nature Based Actions For Heath, Wellbeing & Resilience in Cities (VARCITIES), Horizon 2020-SC5-2019-2 (Grant Agreement number 869505); Italian Ministero della Salute: Eye-movement dynamics during free viewing as biomarker for assessment of visuospatial functions and for closed-loop rehabilitation in stroke (EYEMOVINSTROKE; RF-2019-12369300).

## Conflict of interest

The authors declare that the research was conducted in the absence of any commercial or financial relationships that could be construed as a potential conflict of interest

## Publisher’s note

All claims expressed in this article are solely those of the authors and do not necessarily represent those of their affiliated organizations, or those of the publisher, the editors and the reviewers. Any product that may be evaluated in this article, or claim that may be made by its manufacturer, is not guaranteed or endorsed by the publisher.

## Footnotes

^1^www.itksnap.org
