# Supplementary material for: Patterns of gray and white matter functional networks involvement in glioblastoma patients: indirect mapping from clinical MRI scans
Source: Front Neurol. 2023 Jun 20;14:1175576. doi: 10.3389/fneur.2023.1175576 (PMC10318144; doi:10.3389/fneur.2023.1175576)
Supplement: Supplementary file 1 [file Data_Sheet_1.docx]

Supplementary Material

**Supplementary tables**

| **Supplementary table 1:** significant Bonferroni-corrected post-hoc comparisons between percentages of overlap with GBM core | | | | |
| --- | --- | --- | --- | --- |
| **CORE** | | | | |
| **Significant comparisons between functional networks** | | **Difference** | **Standardized difference** | **p-value** |
| **Gray matter networks** | **Somatomotor B vs Control network C** | 10.5% | 6.819 | <0.0001 |
|  | **Somatomotor B vs Limbic B** | 9.9% | 6.421 | <0.0001 |
|  | **Somatomotor B vs Default Mode Network C** | 9.2% | 5.960 | <0.0001 |
|  | **Somatomotor B vs Peripheral Vision** | 8.5% | 5.523 | <0.0001 |
|  | **Somatomotor B vs Dorsal Attention A** | 7.3% | 4.769 | <0.0001 |
|  | **Somatomotor B vs Dorsal Attention B** | 8.1% | 5.251 | <0.0001 |
|  | **Somatomotor B vs Central Vision** | 8.5% | 5.509 | <0.0001 |
|  | **Somatomotor B vs Somatomotor A** | 7.0% | 4.568 | <0.0001 |
|  | **Somatomotor B vs Control network B** | 7.1% | 4.645 | <0.0001 |
|  | **Somatomotor B vs Temporo-Parietal** | 6.8% | 4.419 | <0.0001 |
|  | **Somatomotor B vs Default Mode Network A** | 5.9% | 3.842 | 0.0002 |
|  | **Somatomotor B vs Limbic A** | 6.2% | 4.051 | <0.0001 |
|  | **Salient Ventral Attention A vs Control network C** | 9.8% | 6.376 | <0.0001 |
|  | **Salient Ventral Attention A vs Limbic B** | 9.2% | 5.978 | <0.0001 |
|  | **Salient Ventral Attention A vs Default Mode Network C** | 8.5% | 5.518 | <0.0001 |
|  | **Salient Ventral Attention A vs Peripheral Vision** | 7.8% | 5.081 | <0.0001 |
|  | **Salient Ventral Attention A vs Dorsal Attention A** | 6.6% | 4.326 | <0.0001 |
|  | **Salient Ventral Attention A vs Dorsal Attention B** | 7.4% | 4.808 | <0.0001 |
|  | **Salient Ventral Attention A vs Central Vision** | 7.8% | 5.066 | <0.0001 |
|  | **Salient Ventral Attention A vs Somatomotor A** | 6.3% | 4.125 | <0.0001 |
|  | **Salient Ventral Attention A vs Control network B** | 6.5% | 4.202 | <0.0001 |
|  | **Salient Ventral Attention A vs Temporo-Parietal** | 6.1% | 3.976 | 0.0003 |
|  | **Salient Ventral Attention A vs Limbic A** | 5.5% | 3.609 | 0.0003 |
|  | **Default Mode Network B vs Control network C** | 7.8% | 5.084 | <0.0001 |
|  | **Default Mode Network B vs Limbic B** | 7.2% | 4.686 | <0.0001 |
|  | **Default Mode Network B vs Default Mode Network C** | 6.5% | 4.225 | <0.0001 |
|  | **Default Mode Network B vs Peripheral Vision** | 5.8% | 3.788 | 0.0002 |
|  | **Default Mode Network B vs Central Vision** | 5.8% | 3.774 | 0.0003 |
|  | **Salient Ventral Attention B vs Control network C** | 6.6% | 4.309 | <0.0001 |
|  | **Salient Ventral Attention B vs Limbic B** | 6.0% | 3.911 | 0.0002 |
|  | **Control network A vs Control network C** | 6.3% | 4.107 | <0.0001 |
| For GMNs, the Bonferroni-corrected significance level was 0.00036 | | | | |
| **White matter networks** | **N12 (Ventral frontoparietal tracts) vs N9 (Posterior cerebellar tracts)** | 16.7% | 8.542 | <0.0001 |
|  | **N12 (Ventral frontoparietal tracts) vs N1 (Cingulum and associated tracts)** | 12.2% | 6.243 | <0.0001 |
|  | **N12 (Ventral frontoparietal tracts) vs N6 (Visual superficial white-matter system)** | 11.6% | 5.927 | <0.0001 |
|  | **N12 (Ventral frontoparietal tracts) vs N4 (Forceps minor system)** | 11.9% | 6.085 | <0.0001 |
|  | **N12 (Ventral frontoparietal tracts) vs N10 (Dorsal frontoparietal tracts)** | 11.1% | 5.674 | <0.0001 |
|  | **N12 (Ventral frontoparietal tracts) vs N3 (Sensorimotor superficial white-matter system)** | 8.0% | 4.099 | <0.0001 |
|  | **N12 (Ventral frontoparietal tracts) vs N2 (Uncinate and middle temporal lobe tracts)** | 8.9% | 4.557 | <0.0001 |
|  | **N12 (Ventral frontoparietal tracts) vs N8 (Inferior corticospinal Tract)** | 8.9% | 4.551 | <0.0001 |
|  | **N5 (Superior longitudinal fasciculus system) vs N9 (Posterior cerebellar tracts)** | 13.2% | 6.746 | <0.0001 |
|  | **N5 (Superior longitudinal fasciculus system) vs N1 (Cingulum and associated tracts)** | 8.7% | 4.447 | <0.0001 |
|  | **N5 (Superior longitudinal fasciculus system) vs N6 (Visual superficial white-matter system)** | 8.1% | 4.131 | <0.0001 |
|  | **N5 (Superior longitudinal fasciculus system) vs N4 (Forceps minor system)** | 8.4% | 4.289 | <0.0001 |
|  | **N5 (Superior longitudinal fasciculus system) vs N10 (Dorsal frontoparietal tracts)** | 7.6% | 3.877 | <0.0001 |
|  | **N11 (Deep frontal white matter) vs N9 (Posterior cerebellar tracts)** | 11.9% | 6.110 | <0.0001 |
|  | **N11 (Deep frontal white matter) vs N1 (Cingulum and associated tracts)** | 7.4% | 3.811 | <0.0001 |
|  | **N11 (Deep frontal white matter) vs N6 (Visual superficial white-matter system)** | 6.8% | 3.495 | 0.0005 |
|  | **N11 (Deep frontal white matter) vs N4 (Forceps minor system)** | 7.1% | 3.653 | 0.0003 |
|  | **N7 (Inferior longitudinal fasciculus system) vs N9 (Posterior cerebellar tracts)** | 11.2% | 5.717 | <0.0001 |
|  | **N7 (Inferior longitudinal fasciculus system) vs N1 (Cingulum and associated tracts)** | 6.7% | 3.418 | 0.0005 |
|  | **N8 (Inferior corticospinal Tract) vs N9 (Posterior cerebellar tracts)** | 7.8% | 3.991 | <0.0001 |
|  | **N2 (Uncinate and middle temporal lobe tracts) vs N9 (Posterior cerebellar tracts)** | 7.8% | 3.985 | <0.0001 |
|  | **N3 (Sensorimotor superficial white-matter system) vs N9 (Posterior cerebellar tracts)** | 8.7% | 4.443 | 0.0001 |
|  | **N3 (Sensorimotor superficial white-matter system) vs N9 (Posterior cerebellar tracts)** | 8.7% | 4.443 | 0.0001 |
|  | **N3 (Sensorimotor superficial white-matter system) vs N9 (Posterior cerebellar tracts)** | 8.7% | 4.443 | 0.0001 |
|  | **N3 (Sensorimotor superficial white-matter system) vs N9 (Posterior cerebellar tracts)** | 8.7% | 4.443 | 0.0001 |
| For WMNs, the Bonferroni-corrected significance level was 0.00075. | | | | |

| **Supplementary table 2:** significant Bonferroni-corrected post-hoc comparisons between percentages of overlap with GBM edema | | | | |
| --- | --- | --- | --- | --- |
| **EDEMA** | | | | |
| **Significant comparison between functional networks** | | **Difference** | **Standardized difference** | **p-value** |
| **Gray matter networks** | **Somatomotor B vs Control network C** | 13.3% | 10.874 | <0.0001 |
|  | **Somatomotor B vs Default Mode Network C** | 13.2% | 10.744 | <0.0001 |
|  | **Somatomotor B vs Limbic B** | 13.1% | 10.714 | <0.0001 |
|  | **Somatomotor B vs Peripheral Vision** | 12.0% | 9.813 | <0.0001 |
|  | **Somatomotor B vs Limbic A** | 12.0% | 9.789 | <0.0001 |
|  | **Somatomotor B vs Central Vision** | 11.3% | 9.197 | <0.0001 |
|  | **Somatomotor B vs Dorsal Attention A** | 9.5% | 7.778 | <0.0001 |
|  | **Somatomotor B vs Somatomotor A** | 8.9% | 7.234 | <0.0001 |
|  | **Somatomotor B vs Temporo-Parietal** | 8.7% | 7.117 | <0.0001 |
|  | **Somatomotor B vs Dorsal Attention B** | 7.9% | 6.443 | <0.0001 |
|  | **Somatomotor B vs Salient Ventral Attention B** | 7.8% | 6.348 | <0.0001 |
|  | **Somatomotor B vs Control Network B** | 7.7% | 6.292 | <0.0001 |
|  | **Somatomotor B vs Default Mode Network A** | 7.5% | 6.087 | <0.0001 |
|  | **Somatomotor B vs Default Mode Network B** | 6.6% | 5.366 | <0.0001 |
|  | **Somatomotor B vs Control Network A** | 4.6% | 3.760 | 0.0002 |
|  | **Salient Ventral Attention A vs Control Network C** | 9.0% | 7.311 | <0.0001 |
|  | **Salient Ventral Attention A vs Default Mode Network C** | 8.8% | 7.182 | <0.0001 |
|  | **Salient Ventral Attention A vs Limbic B** | 8.8% | 7.151 | <0.0001 |
|  | **Salient Ventral Attention A vs Peripheral Vision** | 7.7% | 6.250 | <0.0001 |
|  | **Salient Ventral Attention A vs Limbic A** | 7.6% | 6.226 | <0.0001 |
|  | **Salient Ventral Attention A vs Central Vision** | 6.9% | 5.635 | <0.0001 |
|  | **Salient Ventral Attention A vs Dorsal Attention A** | 5.2% | 4.215 | <0.0001 |
|  | **Salient Ventral Attention A vs Somatomotor A** | 4.5% | 3.671 | 0.00029 |
|  | **Control Network A vs Control Network C** | 8.7% | 7.114 | <0.0001 |
|  | **Control Network A vs Default Mode Network C** | 8.6% | 6.985 | <0.0001 |
|  | **Control Network A vs Limbic B** | 8.5% | 6.954 | <0.0001 |
|  | **Control Network A vs Peripheral Vision** | 7.4% | 6.053 | <0.0001 |
|  | **Control Network A vs Limbic A** | 7.4% | 6.029 | <0.0001 |
|  | **Control Network A vs Central Vision** | 6.7% | 5.438 | <0.0001 |
|  | **Control Network A vs Dorsal Attention A** | 4.9% | 4.018 | 0.0002 |
|  | **Default Mode Network B vs Control Network C** | 6.8% | 5.508 | <0.0001 |
|  | **Default Mode Network B vs Default Mode Network C** | 6.6% | 5.378 | <0.0001 |
|  | **Default Mode Network B vs Limbic B** | 6.6% | 5.348 | <0.0001 |
|  | **Default Mode Network B vs Peripheral Vision** | 5.5% | 4.447 | <0.0001 |
|  | **Default Mode Network B vs Limbic A** | 5.4% | 4.423 | <0.0001 |
|  | **Default Mode Network B vs Central Vision** | 4.7% | 3.831 | 0.000q |
|  | **Default Mode Network A vs Control Network C** | 5.9% | 4.786 | <0.0001 |
|  | **Default Mode Network A vs Default Mode Network C** | 5.7% | 4.657 | <0.0001 |
|  | **Default Mode Network A vs Limbic B** | 5.7% | 4.626 | <0.0001 |
|  | **Default Mode Network A vs Peripheral Vision** | 4.6% | 3.725 | 0.0002 |
|  | **Default Mode Network A vs Limbic A** | 4.5% | 3.701 | 0.0002 |
|  | **Control Network B vs Control Network C** | 5.624 | 4.582 | <0.0001 |
|  | **Control Network B vs Default Mode Network C** | 5.465 | 4.453 | <0.0001 |
|  | **Control Network B vs Limbic B** | 5.427 | 4.422 | <0.0001 |
|  | **Salient Ventral Attention B vs Control Network C** | 5.6% | 4.526 | <0.0001 |
|  | **Salient Ventral Attention B vs Default Mode Network C** | 5.4% | 4.396 | <0.0001 |
|  | **Salient Ventral Attention B vs Limbic B** | 5.4% | 4.366 | <0.0001 |
|  | **Dorsal Attention B vs Control Network C** | 5.438 | 4.431 | <0.0001 |
|  | **Dorsal Attention B vs Default Mode Network C** | 5.279 | 4.301 | <0.0001 |
|  | **Dorsal Attention B vs Limbic B** | 5.241 | 4.271 | <0.0001 |
|  | **Temporo-Parietal vs Control Network C** | 4.610 | 3.756 | 0.0002 |
| For GMNs, the Bonferroni-corrected significance level was 0.00036 | | | | |
| **White matter networks** | **N5 (Superior longitudinal fasciculus system) vs N9 (Posterior cerebellar tracts)** | 23.9% | 16.166 | <0.0001 |
|  | **N5 (Superior longitudinal fasciculus system) vs N8 (Inferior corticospinal Tract)** | 21.5% | 14.519 | <0.0001 |
|  | **N5 (Superior longitudinal fasciculus system) vs N6 (Visual superficial white-matter system)** | 19.9% | 13.420 | <0.0001 |
|  | **N5 (Superior longitudinal fasciculus system) vs N1 (Cingulum and associated tracts)** | 20.0% | 13.536 | <0.0001 |
|  | **N5 (Superior longitudinal fasciculus system) vs N2 (Uncinate and middle temporal lobe tracts)** | 19.1% | 12.878 | <0.0001 |
|  | **N5 (Superior longitudinal fasciculus system) vs N4 (Forceps minor system)** | 19.1% | 12.887 | <0.0001 |
|  | **N5 (Superior longitudinal fasciculus system) vs N3 (Sensorimotor superficial white-matter system)** | 16.0% | 10.807 | <0.0001 |
|  | **N5 (Superior longitudinal fasciculus system) vs N7 (Inferior longitudinal fasciculus system)** | 13.6% | 9.203 | <0.0001 |
|  | **N5 (Superior longitudinal fasciculus system) vs N10 (Dorsal frontoparietal tracts)** | 13.3% | 8.976 | <0.0001 |
|  | **N5 (Superior longitudinal fasciculus system) vs N11 (Deep frontal white matter)** | 12.0% | 8.091 | <0.0001 |
|  | **N5 (Superior longitudinal fasciculus system) vs N12 (Ventral frontoparietal tracts)** | 9.2% | 6.189 | <0.0001 |
|  | **N12 (Ventral frontoparietal tracts) vs N9 (Posterior cerebellar tracts)** | 14.8% | 9.977 | <0.0001 |
|  | **N12 (Ventral frontoparietal tracts) vs N8 (Inferior corticospinal Tract)** | 12.3% | 8.330 | <0.0001 |
|  | **N12 (Ventral frontoparietal tracts) vs N6 (Visual superficial white-matter system)** | 10.7% | 7.231 | <0.0001 |
|  | **N12 (Ventral frontoparietal tracts) vs N1 (Cingulum and associated tracts)** | 10.9% | 7.346 | <0.0001 |
|  | **N12 (Ventral frontoparietal tracts) vs N2 (Uncinate and middle temporal lobe tracts)** | 9.9% | 6.689 | <0.0001 |
|  | **N12 (Ventral frontoparietal tracts) vs N4 (Forceps minor system)** | 9.9% | 6.698 | <0.0001 |
|  | **N12 (Ventral frontoparietal tracts) vs N3 (Sensorimotor superficial white-matter system)** | 6.8% | 4.618 | <0.0001 |
|  | **N11 (Deep frontal white matter) vs N9 (Posterior cerebellar tracts)** | 12.0% | 8.076 | <0.0001 |
|  | **N11 (Deep frontal white matter) vs N8 (Inferior corticospinal Tract)** | 9.5% | 6.429 | <0.0001 |
|  | **N11 (Deep frontal white matter) vs N6 (Visual superficial white-matter system)** | 7.9% | 5.330 | <0.0001 |
|  | **N11 (Deep frontal white matter) vs N1 (Cingulum and associated tracts)** | 8.1% | 5.445 | <0.0001 |
|  | **N11 (Deep frontal white matter) vs N2 (Uncinate and middle temporal lobe tracts)** | 7.1% | 4.787 | <0.0001 |
|  | **N11 (Deep frontal white matter) vs N4 (Forceps minor system)** | 7.1% | 4.797 | <0.0001 |
|  | **N10 (Dorsal frontoparietal tracts) vs N9 (Posterior cerebellar tracts)** | 10.6% | 7.190 | <0.0001 |
|  | **N10 (Dorsal frontoparietal tracts) vs N8 (Inferior corticospinal Tract)** | 8.2% | 5.543 | <0.0001 |
|  | **N10 (Dorsal frontoparietal tracts) vs N6 (Visual superficial white-matter system)** | 6.6% | 4.444 | <0.0001 |
|  | **N10 (Dorsal frontoparietal tracts) vs N1 (Cingulum and associated tracts)** | 6.8% | 4.560 | <0.0001 |
|  | **N10 (Dorsal frontoparietal tracts) vs N4 (Forceps minor system)** | 5.8% | 3.911 | <0.0001 |
|  | **N10 (Dorsal frontoparietal tracts) vs N2 (Uncinate and middle temporal lobe tracts)** | 5.8% | 3.902 | 0.00010 |
|  | **N7 (Inferior longitudinal fasciculus system) vs N9 (Posterior cerebellar tracts)** | 10.3% | 6.963 | <0.0001 |
|  | **N7 (Inferior longitudinal fasciculus system) vs N8 (Inferior corticospinal Tract)** | 7.9% | 5.316 | <0.0001 |
|  | **N7 (Inferior longitudinal fasciculus system) vs N6 (Visual superficial white-matter system)** | 6.2% | 4.217 | 0.0001 |
|  | **N7 (Inferior longitudinal fasciculus system) vs N1 (Cingulum and associated tracts)** | 6.4% | 4.332 | 0.0003 |
|  | **N7 (Inferior longitudinal fasciculus system) vs N4 (Forceps minor system)** | 5.5% | 3.684 | 0.00024 |
|  | **N7 (Inferior longitudinal fasciculus system) vs N2 (Uncinate and middle temporal lobe tracts)** | 5.4% | 3.675 | 0.00025 |
|  | **N3 (Sensorimotor superficial white-matter system) vs N9 (Posterior cerebellar tracts)** | 7.9% | 5.359 | <0.0001 |
|  | **N3 (Sensorimotor superficial white-matter system) vs N8 (Inferior corticospinal Tract)** | 5.5% | 3.712 | 0.0002 |
| For WMNs, the Bonferroni-corrected significance level was 0.00075. | | | | |

| **Supplementary table 3:** Multiple linear regression models including only network overlap percentages as regressors | | | | | | | | | | | | | | |
| --- | --- | --- | --- | --- | --- | --- | --- | --- | --- | --- | --- | --- | --- | --- |
| **Normalized overlap percentages** | | | | | | | | | | | | | | |
| **Model:** Independent variables or regressors: **percentages of overlap between GBM core and GMNs**  Dependent variable: overall survival (OS) | | | | | | | | **Model:** Independent variables or regressors: **percentages of overlap between GBM core and WMNs**  Dependent variable: overall survival (OS) | | | | | | |
| Model parameters | | Adjusted R^2^ | -0.1 | | | | | Model parameters | Adjusted R^2^ | | 0.017 | | | |
|  |  | F | 0.4 | | | | |  | F | | 1.1 | | | |
|  |  | p | 0.9 (not significant) | | | | |  | p | | 0.3 (not significant) | | | |
| **Model**: Independent variables or regressors: **percentages of overlap between GBM edema and GMNs**  Dependent variable: overall survival (OS) | | | | | | | | **Model:** Independent variables or regressors: **percentages of overlap between GBM edema and WMNs**  Dependent variable: overall survival (OS) | | | | | | |
| Model parameters | | Adjusted R^2^ | -0.07 | | | | | Model parameters | Adjusted R^2^ | 0.12 | | | | |
|  |  | F | 0.6 | | | | |  | F | 2 | | | | |
|  |  | p | 0.8 (not significant) | | | | |  | p | **0.03** | | | | |
|  | | | | | | | | Significant regressors | Variable | N5-edema overlap | | N8-edema overlap | | |
|  |  |  |  |  |  |  |  |  | β | -5 | | -3.2 | | |
|  |  |  |  |  |  |  |  |  | p | 0.03 | | 0.02 | | |
| **Multiple linear regression models including both clinical-prognostic variables and network overlap percentages as regressors** | | | | | | | | | | | | | | |
| **Not normalized overlap percentages*** | | | | | | | | | | | | | | |
| **Model:** Independent variables or regressors: **percentages of overlap between GBM core and GMNs**  Dependent variable: overall survival (OS) | | | | | | | | **Model:** Independent variables or regressors: **percentages of overlap between GBM core and WMNs**  Dependent variable: overall survival (OS) | | | | | | |
| Model parameters | Adjusted R^2^ | | | 0.24 | | | | Model parameters | Adjusted R^2^ | | | 0.31 | | |
|  | F | | | 2.2 | | | |  | F | | | 3.3 | | |
|  | p | | | 0.006 | | | |  | p | | | <0.001 | | |
| Significant regressors | Variable | | | ECOG | MGMT | Somatomotor B | | Significant regressors | Variable | | | ECOG | MGMT | Radicality of surgery |
|  | β | | | -3.3 | 6.7 | 3 | |  | β | | | -4 | 6.5 | 2 |
|  | p | | | 0.014 | 0.007 | 0.049 | |  | p | | | 0.004 | 0.003 | 0.05 |
| **Model**: Independent variables or regressors: **percentages of overlap between GBM edema and GMNs**  Dependent variable: overall survival (OS) | | | | | | | | **Model:** Independent variables or regressors: **percentages of overlap between GBM edema and WMNs**  Dependent variable: overall survival (OS) | | | | | | |
| Model parameters | Adjusted R^2^ | | | 0.19 | | | | Model parameters | Adjusted R^2^ | | | 0.32 | | |
|  | F | | | 1.8 | | | |  | F | | | 3.3 | | |
|  | p | | | 0.03 | | | |  | p | | | <0.001 | | |
| Significant regressors | Variable | | | ECOG | | | | Significant regressors | Variable | | | ECOG | Radicality of surgery | |
|  | β | | | -4.2 | | | |  | β | | | -3.5 | 2.4 | |
|  | p | | | 0.003 | | | |  | p | | | 0.003 | 0.02 | |
| **Alternative overlap percentages (calculated as percentages of network volume overlapped by core/edema)*** | | | | | | | | | | | | | | |
| **Model:** Independent variables or regressors: **percentages of overlap between GBM core and GMNs**  Dependent variable: overall survival (OS) | | | | | | | | **Model:** Independent variables or regressors: **percentages of overlap between GBM core and WMNs**  Dependent variable: overall survival (OS) | | | | | | |
| Model parameters | Adjusted R^2^ | | | 0.26 | | | | Model parameters | Adjusted R^2^ | | | 0.29 | | |
|  | F | | | 2.3 | | | |  | F | | | 3.1 | | |
|  | p | | | 0.003 | | | |  | p | | | <0.001 | | |
| Significant regressors | Variable | | | ECOG | MGMT | Core volume | Somatomotor B | Significant regressors | Variable | | | ECOG | MGMT | |
|  | β | | | -3.4 | 7.1 | -9.9 | 3.6 |  | β | | | -3.8 | 6.4 | |
|  | p | | | 0.008 | 0.002 | 0.046 | 0.045 |  | p | | | 0.004 | 0.003 | |
| **Model**: Independent variables or regressors: **percentages of overlap between GBM edema and GMNs**  Dependent variable: overall survival (OS) | | | | | | | | **Model:** Independent variables or regressors: **percentages of overlap between GBM edema and WMNs**  Dependent variable: overall survival (OS) | | | | | | |
| Model parameters | Adjusted R^2^ | | | 0.25 | | | | Model parameters | Adjusted R^2^ | | | 0.27 | | |
|  | F | | | 2.2 | | | |  | F | | | 2.8 | | |
|  | p | | | 0.007 | | | |  | p | | | 0.001 | | |
| Significant regressors | Variable | | | ECOG | MGMT | Radicality of surgery | | Significant regressors | Variable | | | ECOG | | |
|  | β | | | -4.3 | 5 | 2.4 | |  | β | | | -4.1 | | |
|  | p | | | 0.001 | 0.03 | 0.03 | |  | p | | | 0.001 | | |
| *multiple linear regression models including only not normalized overlap percentages or alternative overlap percentages (without clinical-prognostic variables) are not shown but were all not significant | | | | | | | | | | | | | | |

| **Supplementary table 4:** Correlations between radicality of surgery and network overlap percentages | | | | | |
| --- | --- | --- | --- | --- | --- |
| **Normalized overlap percentages** | | | | | |
| **CORE** | | | **EDEMA** | | |
| **Network** | **R** | **p** | **Network** | **R** | **p** |
| **Central Vision** | -0.12 | 0.25 | **Central Vision** | -0.09 | 0.41 |
| **Peripheral Vision** | -0.12 | 0.25 | **Peripheral Vision** | -0.09 | 0.40 |
| **Somatomotor A** | 0.05 | 0.60 | **Somatomotor A** | 0.02 | 0.88 |
| **Somatomotor B** | 0.23 | 0.03 | **Somatomotor B** | 0.24 | 0.02 |
| **Dorsal Attention A** | -0.13 | 0.21 | **Dorsal Attention A** | -0.07 | 0.52 |
| **Dorsal Attention B** | -0.12 | 0.25 | **Dorsal Attention B** | 0.05 | 0.65 |
| **Salient Ventral Attention A** | 0.14 | 0.18 | **Salient Ventral Attention A** | 0.05 | 0.66 |
| **Salient Ventral Attention B** | -0.01 | 0.94 | **Salient Ventral Attention B** | 0.02 | 0.86 |
| **Limbic A** | -0.06 | 0.54 | **Limbic A** | -0.17 | 0.11 |
| **Limbic B** | -0.19 | 0.07 | **Limbic B** | 0.00 | 1.00 |
| **Control network A** | -0.05 | 0.61 | **Control network A** | -0.03 | 0.80 |
| **Control network B** | -0.08 | 0.43 | **Control network B** | -0.13 | 0.22 |
| **Control network C** | -0.07 | 0.52 | **Control network C** | -0.11 | 0.33 |
| **Default Mode Network A** | -0.01 | 0.92 | **Default Mode Network A** | -0.15 | 0.16 |
| **Default Mode Network B** | 0.24 | 0.02 | **Default Mode Network B** | -0.05 | 0.64 |
| **Default Mode Network C** | -0.14 | 0.18 | **Default Mode Network C** | -0.11 | 0.31 |
| **Temporo-Parietal** | 0.05 | 0.64 | **Temporo-Parietal** | 0.18 | 0.09 |
| **Subcortical gray matter (basal ganglia and thalami)** | -0.10 | 0.36 | **Subcortical gray matter (basal ganglia and thalami)** | -0.01 | 0.95 |
| **Hippocampi** | -0.19 | 0.08 | **Hippocampi** | 0.16 | 0.13 |
| **N1 (Cingulum and associated tracts)** | 0.07 | 0.49 | **N1 (Cingulum and associated tracts)** | 0.01 | 0.91 |
| **N2 (Uncinate and middle temporal lobe tracts)** | 0.08 | 0.45 | **N2 (Uncinate and middle temporal lobe tracts)** | -0.12 | 0.25 |
| **N3 (Sensorimotor superficial white-matter system)** | 0.09 | 0.40 | **N3 (Sensorimotor superficial white-matter system)** | 0.07 | 0.53 |
| **N4 (Forceps minor system)** | -0.05 | 0.64 | **N4 (Forceps minor system)** | -0.17 | 0.11 |
| **N5 (Superior longitudinal fasciculus system)** | -0.12 | 0.26 | **N5 (Superior longitudinal fasciculus system)** | 0.09 | 0.43 |
| **N6 (Visual superficial white-matter system)** | -0.12 | 0.24 | **N6 (Visual superficial white-matter system)** | -0.10 | 0.35 |
| **N7 (Inferior longitudinal fasciculus system)** | -0.04 | 0.71 | **N7 (Inferior longitudinal fasciculus system)** | 0.00 | 1.00 |
| **N8 (Inferior corticospinal Tract)** | -0.04 | 0.69 | **N8 (Inferior corticospinal Tract)** | 0.01 | 0.96 |
| **N9 (Posterior cerebellar tracts)** | -0.09 | 0.38 | **N9 (Posterior cerebellar tracts)** | 0.08 | 0.44 |
| **N10 (Dorsal frontoparietal tracts)** | 0.10 | 0.32 | **N10 (Dorsal frontoparietal tracts)** | 0.09 | 0.40 |
| **N11 (Deep frontal white matter)** | -0.07 | 0.50 | **N11 (Deep frontal white matter)** | -0.13 | 0.22 |
| **N12 (Ventral frontoparietal tracts)** | 0.12 | 0.25 | **N12 (Ventral frontoparietal tracts)** | 0.20 | 0.06 |
| **Not normalized overlap percentages** | | | | | |
| **CORE** | | | **EDEMA** | | |
| **Network** | **R** | **p** | **Network** | **R** | **p** |
| **Central Vision** | -0.11 | 0.27 | **Central Vision** | -0.075 | 0.49 |
| **Peripheral Vision** | -0.11 | 0.29 | **Peripheral Vision** | -0.105 | 0.33 |
| **Somatomotor A** | 0.06 | 0.57 | **Somatomotor A** | 0.020 | 0.85 |
| **Somatomotor B** | 0.25 | 0.02 | **Somatomotor B** | 0.248 | 0.02 |
| **Dorsal Attention A** | -0.11 | 0.30 | **Dorsal Attention A** | -0.072 | 0.50 |
| **Dorsal Attention B** | -0.11 | 0.29 | **Dorsal Attention B** | 0.046 | 0.67 |
| **Salient Ventral Attention A** | 0.21 | 0.04 | **Salient Ventral Attention A** | 0.074 | 0.50 |
| **Salient Ventral Attention B** | 0.03 | 0.81 | **Salient Ventral Attention B** | 0.035 | 0.74 |
| **Limbic A** | -0.03 | 0.77 | **Limbic A** | -0.181 | 0.09 |
| **Limbic B** | -0.16 | 0.13 | **Limbic B** | -0.009 | 0.93 |
| **Control network A** | -0.02 | 0.83 | **Control network A** | -0.012 | 0.91 |
| **Control network B** | -0.07 | 0.48 | **Control network B** | -0.119 | 0.27 |
| **Control network C** | -0.05 | 0.63 | **Control network C** | -0.112 | 0.30 |
| **Default Mode Network A** | 0.01 | 0.96 | **Default Mode Network A** | -0.153 | 0.15 |
| **Default Mode Network B** | 0.26 | 0.01 | **Default Mode Network B** | -0.077 | 0.48 |
| **Default Mode Network C** | -0.11 | 0.27 | **Default Mode Network C** | -0.110 | 0.31 |
| **Temporo-Parietal** | 0.05 | 0.63 | **Temporo-Parietal** | 0.196 | 0.07 |
| **Subcortical gray matter (basal ganglia and thalami)** | -0.07 | 0.50 | **Subcortical gray matter (basal ganglia and thalami)** | -0.009 | 0.93 |
| **Hippocampi** | -0.16 | 0.11 | **Hippocampi** | 0.182 | 0.09 |
| **N1 (Cingulum and associated tracts)** | -0.04 | 0.69 | **N1 (Cingulum and associated tracts)** | -0.007 | 0.95 |
| **N2 (Uncinate and middle temporal lobe tracts)** | 0.03 | 0.80 | **N2 (Uncinate and middle temporal lobe tracts)** | -0.097 | 0.37 |
| **N3 (Sensorimotor superficial white-matter system)** | 0.07 | 0.53 | **N3 (Sensorimotor superficial white-matter system)** | 0.053 | 0.62 |
| **N4 (Forceps minor system)** | -0.01 | 0.90 | **N4 (Forceps minor system)** | -0.168 | 0.12 |
| **N5 (Superior longitudinal fasciculus system)** | -0.11 | 0.31 | **N5 (Superior longitudinal fasciculus system)** | 0.064 | 0.56 |
| **N6 (Visual superficial white-matter system)** | -0.14 | 0.18 | **N6 (Visual superficial white-matter system)** | -0.121 | 0.26 |
| **N7 (Inferior longitudinal fasciculus system)** | -0.08 | 0.43 | **N7 (Inferior longitudinal fasciculus system)** | 0.003 | 0.98 |
| **N8 (Inferior corticospinal Tract)** | -0.12 | 0.24 | **N8 (Inferior corticospinal Tract)** | 0.026 | 0.81 |
| **N9 (Posterior cerebellar tracts)** | -0.10 | 0.34 | **N9 (Posterior cerebellar tracts)** | 0.071 | 0.51 |
| **N10 (Dorsal frontoparietal tracts)** | 0.08 | 0.46 | **N10 (Dorsal frontoparietal tracts)** | 0.075 | 0.49 |
| **N11 (Deep frontal white matter)** | -0.07 | 0.49 | **N11 (Deep frontal white matter)** | -0.121 | 0.26 |
| **N12 (Ventral frontoparietal tracts)** | 0.08 | 0.47 | **N12 (Ventral frontoparietal tracts)** | 0.211 | 0.05 |
| **Alternative network overlap percentage (percentage of network overlapped by core/edema)** | | | | | |
| **CORE** | | | **EDEMA** | | |
| **Network** | **R** | **p** | **Network** | **R** | **p** |
| **Central Vision** | -0.09 | 0.38 | **Central Vision** | -0.06 | 0.59 |
| **Peripheral Vision** | -0.13 | 0.23 | **Peripheral Vision** | -0.11 | 0.29 |
| **Somatomotor A** | 0.01 | 0.92 | **Somatomotor A** | 0.10 | 0.36 |
| **Somatomotor B** | 0.19 | 0.06 | **Somatomotor B** | 0.06 | 0.59 |
| **Dorsal Attention A** | -0.12 | 0.26 | **Dorsal Attention A** | -0.05 | 0.66 |
| **Dorsal Attention B** | -0.15 | 0.16 | **Dorsal Attention B** | 0.08 | 0.46 |
| **Salient Ventral Attention A** | 0.14 | 0.19 | **Salient Ventral Attention A** | 0.08 | 0.46 |
| **Salient Ventral Attention B** | 0.01 | 0.89 | **Salient Ventral Attention B** | 0.06 | 0.61 |
| **Limbic A** | -0.01 | 0.93 | **Limbic A** | -0.12 | 0.26 |
| **Limbic B** | -0.24 | 0.02 | **Limbic B** | 0.09 | 0.41 |
| **Control network A** | -0.09 | 0.40 | **Control network A** | -0.10 | 0.35 |
| **Control network B** | -0.09 | 0.41 | **Control network B** | 0.01 | 0.94 |
| **Control network C** | -0.04 | 0.73 | **Control network C** | -0.01 | 0.91 |
| **Default Mode Network A** | 0.08 | 0.44 | **Default Mode Network A** | 0.06 | 0.59 |
| **Default Mode Network B** | -0.13 | 0.22 | **Default Mode Network B** | -0.10 | 0.35 |
| **Default Mode Network C** | 0.04 | 0.67 | **Default Mode Network C** | -0.05 | 0.65 |
| **Temporo-Parietal** | 0.21 | 0.04 | **Temporo-Parietal** | -0.05 | 0.67 |
| **Subcortical gray matter (basal ganglia and thalami)** | -0.07 | 0.49 | **Subcortical gray matter (basal ganglia and thalami)** | 0.03 | 0.75 |
| **Hippocampi** | -0.13 | 0.21 | **Hippocampi** | 0.10 | 0.38 |
| **N1 (Cingulum and associated tracts)** | -0.13 | 0.23 | **N1 (Cingulum and associated tracts)** | 0.03 | 0.79 |
| **N2 (Uncinate and middle temporal lobe tracts)** | 0.02 | 0.88 | **N2 (Uncinate and middle temporal lobe tracts)** | -0.12 | 0.27 |
| **N3 (Sensorimotor superficial white-matter system)** | -0.02 | 0.83 | **N3 (Sensorimotor superficial white-matter system)** | 0.15 | 0.16 |
| **N4 (Forceps minor system)** | 0.06 | 0.60 | **N4 (Forceps minor system)** | -0.12 | 0.25 |
| **N5 (Superior longitudinal fasciculus system)** | -0.17 | 0.10 | **N5 (Superior longitudinal fasciculus system)** | 0.09 | 0.39 |
| **N6 (Visual superficial white-matter system)** | -0.13 | 0.21 | **N6 (Visual superficial white-matter system)** | -0.10 | 0.34 |
| **N7 (Inferior longitudinal fasciculus system)** | -0.10 | 0.36 | **N7 (Inferior longitudinal fasciculus system)** | -0.06 | 0.58 |
| **N8 (Inferior corticospinal Tract)** | -0.10 | 0.32 | **N8 (Inferior corticospinal Tract)** | -0.09 | 0.43 |
| **N9 (Posterior cerebellar tracts)** | -0.07 | 0.50 | **N9 (Posterior cerebellar tracts)** | 0.06 | 0.60 |
| **N10 (Dorsal frontoparietal tracts)** | -0.08 | 0.43 | **N10 (Dorsal frontoparietal tracts)** | 0.11 | 0.32 |
| **N11 (Deep frontal white matter)** | -0.03 | 0.77 | **N11 (Deep frontal white matter)** | -0.07 | 0.53 |
| **N12 (Ventral frontoparietal tracts)** | -0.01 | 0.96 | **N12 (Ventral frontoparietal tracts)** | 0.07 | 0.53 |
| All the above reported correlations were not statistically significant after Bonferroni correction for multiple comparisons.  Bonferroni corrected significant level alpha for correlations with GMN overlap percentages: 0.0026; for correlations with WMN overlap percentages: 0.004 | | | | | |

**Supplementary Figures**

**
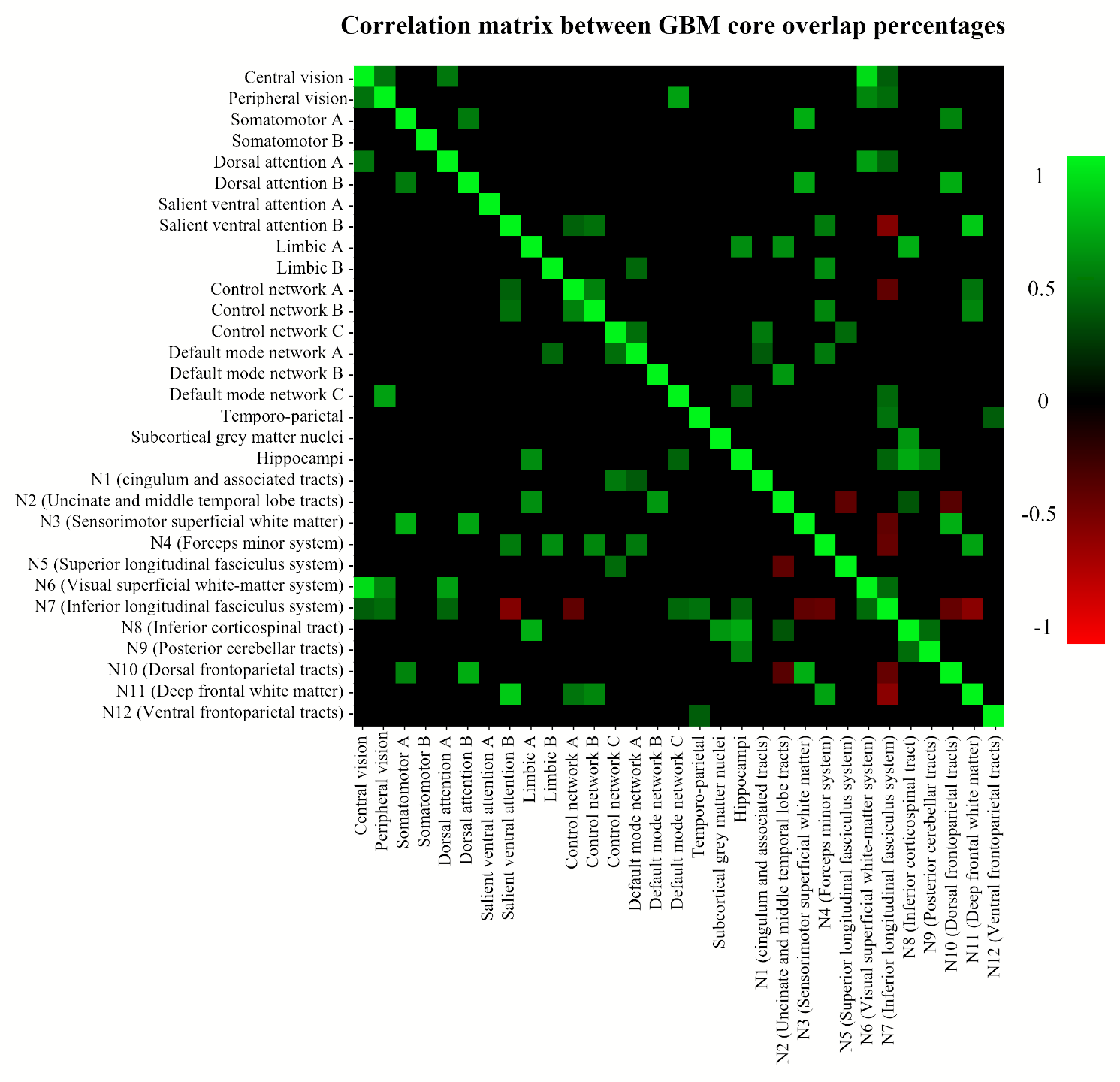
**

**Supplementary figure 1.** Correlation matrix between overlap percentages referred to GBM core; each cell represents the corresponding Pearson’s correlation coefficient R. Only correlations that remained statistically significant after Bonferroni correction for multiple comparisons are shown in a color scale from red (negative R), to black (no linear correlation), to green (positive R); non-significant correlations are all shown in black.

**
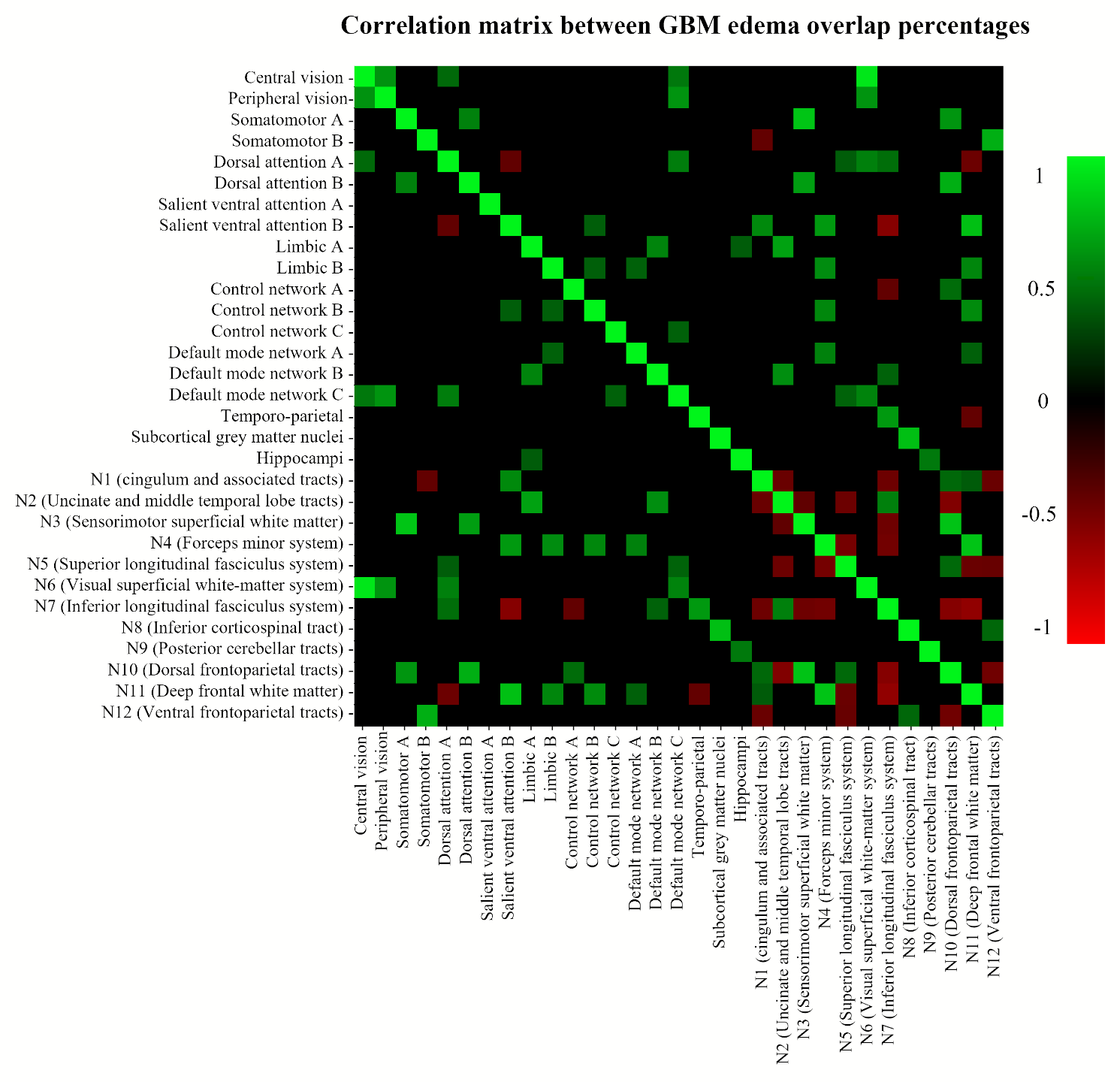
**

**Supplementary figure 2.** Correlation matrix between overlap percentages referred to GBM edema; each cell represents the corresponding Pearson’s correlation coefficient R. Only correlations that remained statistically significant after Bonferroni correction for multiple comparisons are shown in a color scale from red (negative R), to black (no linear correlation), to green (positive R); non-significant correlations are all shown in black.
